# Tailoring the lineshapes of coupled plasmonic systems based on a theory derived from first principles

**DOI:** 10.1038/s41377-020-00386-5

**Published:** 2020-09-08

**Authors:** Jing Lin, Meng Qiu, Xiyue Zhang, Huijie Guo, Qingnan Cai, Shiyi Xiao, Qiong He, Lei Zhou

**Affiliations:** 1 State Key Laboratory of Surface Physics, Key Laboratory of Micro and Nano Photonic Structures (Ministry of Education) and Department of Physics, Fudan University, Shanghai, 200433 China; 2 Key Laboratory of Specialty Fiber Optics and Optical Access Networks, Joint International Research Laboratory of Specialty Fiber Optics and Advanced Communication, Shanghai Institute for Advanced Communication and Data Science, Shanghai University, Shanghai, 200444 China; 3 Collaborative Innovation Center of Advanced Microstructures, Nanjing, 210093 China

**Keywords:** Nanophotonics and plasmonics, Sub-wavelength optics, Nanoparticles

## Abstract

Coupled photonic systems exhibit intriguing optical responses attracting intensive attention, but available theoretical tools either cannot reveal the underlying physics or are empirical in nature. Here, we derive a rigorous theoretical framework from first principles (i.e., Maxwell’s equations), with all parameters directly computable via wave function integrations, to study coupled photonic systems containing multiple resonators. Benchmark calculations against Mie theory reveal the physical meanings of the parameters defined in our theory and their mutual relations. After testing our theory numerically and experimentally on a realistic plasmonic system, we show how to utilize it to freely tailor the lineshape of a coupled system, involving two plasmonic resonators exhibiting arbitrary radiative losses, particularly how to create a completely “dark” mode with vanishing radiative loss (e.g., a bound state in continuum). All theoretical predictions are quantitatively verified by our experiments at near-infrared frequencies. Our results not only help understand the profound physics in such coupled photonic systems, but also offer a powerful tool for fast designing functional devices to meet diversified application requests.

## Plasmonics: developing a theory for coupled photonic systems

A new theoretical framework can help clarify the underlying physics of coupled photonic systems whose interacting oscillating particles lead to interesting applications in nanolasing and information transport. The theory, developed by Lei Zhou of Fudan University and colleagues in China, is based on foundational Maxwell’s equations and capable to predict the optical lineshapes of arbitrarily coupled systems. The team tested their theory in simulations and an experiment where near-infrared light was shone on specially placed and shaped nanostructures, obtaining the expected spectroscopic results. They also used it to choose a specific coupling between two nanostructures that achieves a bound state in continuum: waves that remain localized despite coexisting with radiating waves that can carry their energy away. The scientists hope the theory can help improve the theoretical understanding of coupled photonic systems, which has so far been lacking.

## Introduction

Recently, photonic systems consisting of multiple plasmonic/dielectric resonators coupled in different ways have attracted much attention^1–4^. Compared to simple systems containing only one type of resonators, coupled systems exhibit more fascinating near-field (NF) properties (e.g., local field enhancement) and far-field (FF) responses manifested by unusual lineshapes, such as Fano resonance^5–7^ and Rabi oscillations^8,9^, dictated ultimately by how the involved resonators are coupled together. Couplings have offered more opportunities for controlling the NF and FF light environments of such complex photonic systems as desired, making them particularly useful in applications, such as nanolasing^10,11^, fluorescence enhancement^12–14^, and information transport^15–17^.

**Fig. 1:**
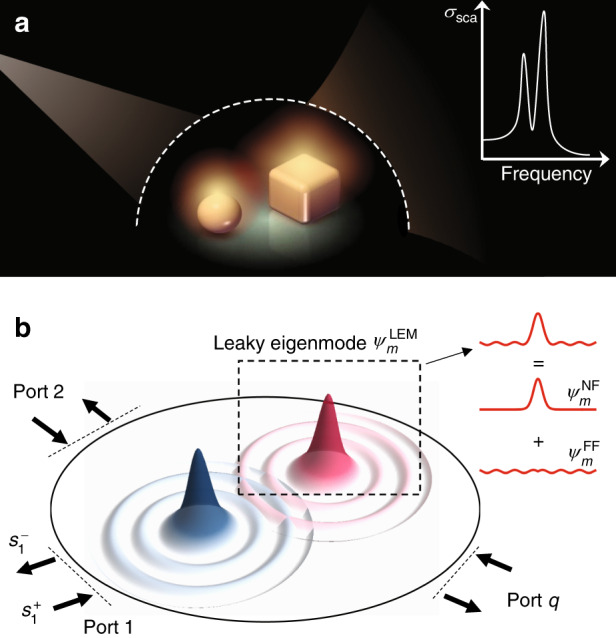
Schematics of the system under study and our theory. **a** Photonic system containing multiple arbitrary resonators coupled together under external illumination. The inset shows a typical optical lineshape of such a system. **b** Schematics of our theory: under certain external illumination, the total scattered field of the coupled system is a linear combination of leaky eigenmodes (LEM, $\psi _m^{{\mathrm{LEM}}}$) of different resonators, each containing a near-field part $\psi _m^{{\mathrm{NF}}}$ and a far-field tail $\psi _m^{{\mathrm{FF}}}$

Despite great advances on the experimental side, the theoretical understandings of such systems are far from satisfactory, which also hinders the rapid design of appropriate systems with desired NF and FF responses. For example, full-wave simulations require huge computing costs and reveal very little physics. Meanwhile, although many models (e.g., coupled-mode theory (CMT)^18–21^, Fano’s formula^22,23^, or effective circuit models^24,25^) were proposed to analyze the underlying physics, they typically require model parameters fitted from simulation results, and thus cannot predict unknown phenomena before having studied the systems numerically. As an early attempt, a photonic tight-binding method (TBM)^26^, with all involved parameters computable without fitting procedures, was proposed to successfully predict the resonance peak positions of a coupled system. Unfortunately, the TBM provides no information on the entire optical responses (e.g., the lineshapes), which are usually more desired for practical applications. The intrinsic difficulties are that these systems are open in nature, in which different resonators can couple not only with each other via NFs but also, more importantly, with external free space via FF interactions (Fig. 1a). To establish a complete theory to predict the entire optical properties of arbitrarily coupled photonic systems, one needs to rigorously consider both NF and FF interactions on the same foot. While several semi-analytical approaches have recently appeared, they have their own limitations and are not generic enough to study arbitrarily coupled systems in a formal way^27–29^.

In this paper, we derive a formal theoretical framework from first principles (i.e., Maxwell’s equations), with all involved parameters directly computable without fitting procedures, to predict the optical lineshapes of arbitrarily coupled photonic systems. The obtained equations resemble the empirical CMT but are derived from first principles, and thus have unambiguous physical meanings, as clearly revealed by benchmark calculations against rigorous Mie theory on a model system. After validating our theory through comparison with experimental/numerical results on a realistic plasmonic metasurface, we present how to employ it to tailor the lineshape of a coupled plasmonic system as desired by varying the interresonator coupling. In particular, we show that it is possible to generate a completely “dark” optical mode with vanishing radiative loss (i.e., a bound state in continuum (BIC)^30,31^) in such systems, although the constituent resonators exhibit moderate radiative losses. All theoretical predictions are quantitatively verified by experimental results on a series of metasurfaces containing plasmonic resonators coupled in different ways.

## Results

### Establishment of the formal theory

We start by establishing a formal theory applicable to generic coupled open systems. As shown in Fig. 1a, we consider the scatterings of a system consisting of *M* arbitrary resonators located at different positions in a host medium under certain external illumination. Such an open system can be schematically described by the model depicted in Fig. 1b, where the region containing resonators is connected to the external continuum via *N* ports with well-defined properties. Formally, we need to solve the following Schrödinger-like equation:

1 $$\widehat {\mathbf{{{H}}}}{\Psi} (\vec r,\omega ) = \omega {\Psi} (\vec r,\omega )$$

where ${\Psi} (\vec r,\omega )$ is the total wave function, and $\widehat {\mathbf{{{H}}}} = \widehat {\mathbf{{{H}}}}_{\mathbf{h}} + \mathop {\sum}\nolimits_m {\widehat {\mathbf{V}}_m}$ is the Hamiltonian of the whole system with $\widehat {\mathbf{{{H}}}}_{\mathbf{h}}$ describing the host medium and $\widehat {\mathbf{V}}_m$ the potential contributed by the *m*th resonator.

To expand the unknown function ${\Psi} (\vec r,\omega )$ appropriately, we need a complete set of basis wave functions that are orthogonal to each other and normalizable in certain ways. In the same spirit as the TBM^26,32^, here, we define a set of wave functions $\{ \psi _{^m}^{{\mathrm{LEM}}}(\vec r,\omega ),m = 1,...,M\}$, which are the (approximate) solutions of the Hamiltonian $\widehat {\mathbf{{{H}}}}_m = \widehat {\mathbf{{{H}}}}_{\mathbf{h}} + \widehat {\mathbf{V}}_m$ (with eigenvalue *ω*), describing the subsystem containing only the *m*th resonator. For simplicity, here, we assume that each resonator supports only one mode, and the extensions to more general cases (e.g., resonators exhibiting multiple or degenerate modes) are straightforward. Different from the systems treated by the TBM, which are closed^26^, and thus have well-defined localized eigenfunctions, here, the open systems under study only support leaky eigenmodes (LEM), as explained subsequently.

Suppose that the resonators exhibit high quality (*Q*) factors; we can use the following approach to obtain $\psi _{^m}^{{\mathrm{LEM}}}(\vec r,\omega )$. Shining the subsystem with external illumination, we can solve $\widehat {\mathbf{{{H}}}}_m{\Psi} _m = \omega {\Psi} _m$ to obtain Ψ_*m*_ analytically or numerically, and then obtain the response spectrum of the system. We then identify the resonance frequency *ω*_*m*_ of the *m*th resonator from the maximum of the response spectrum. Choosing a “background” representing the system at a frequency far from all resonances, we can calculate the background wave function Ψ_B_ by shining the “background” medium with the same external illumination. We finally obtain the desired LEM wave function through $\psi _{^m}^{{\mathrm{LEM}}} = {\Psi} _m - {\Psi} _{\mathrm{B}}$ for the *m*th resonator. We note that $\{ \psi _{^m}^{{\mathrm{LEM}}}\}$ are quite different from the quasi-normal-mode (QNM) functions defined in refs. ^27,28,33^. While $\{ \psi _{^m}^{{\mathrm{LEM}}}\}$are wave functions of the systems under external illumination at real frequencies *ω*_*m*_, QNM functions are eigenfunctions of the systems without external illumination corresponding to complex eigenfrequencies. Moreover, LEM functions do not diverge at infinity, whereas QNM functions inevitably diverge^34,35^. Therefore, LEM functions are particularly suitable for the lineshape problems studied here, which require external illumination. Examples of how to obtain $\psi _{^m}^{{\mathrm{LEM}}}$ and detailed comparisons between LEM and QNM are presented in Sec. I of the Supplementary Information.

Before proceeding further, we first discuss the properties of $\psi _{^m}^{{\mathrm{LEM}}}(\vec r,\omega _m)$. Due to nonnegligible radiation in open systems, $\psi _{^m}^{{\mathrm{LEM}}}(\vec r,\omega _m)$ must contain an FF tail propagating to the external continuum (see the inset in Fig. 1b), making $\psi _{^m}^{{\mathrm{LEM}}}(\vec r,\omega _m)$ un-normalizable within the whole space. To solve this issue, we purposely reexpress $\psi _{^m}^{{\mathrm{LEM}}}(\vec r,\omega _m)$ as

2 $$\psi _m^{{\mathrm{LEM}}}(\vec r,\omega _m) = \psi _m^{{\mathrm{NF}}}(\vec r,\omega _m) + \psi _m^{{\mathrm{FF}}}(\vec r,\omega _m)$$

where $\psi _m^{{\mathrm{NF}}}$ and $\psi _m^{{\mathrm{FF}}}$ represent the NF and FF parts of the wave function, respectively. Technically, for any given system with well-defined external ports, we can always project $\psi _m^{{\mathrm{LEM}}}$ onto the port modes on reference planes of all external ports and then construct $\psi _m^{{\mathrm{FF}}}$ by these port modes, which are assumed to fill the entire space. With $\psi _m^{{\mathrm{FF}}}$ known, we then obtain $\psi _m^{{\mathrm{NF}}}$ numerically based on Eq. (2).

The NF functions $\psi _m^{{\mathrm{NF}}}$ have good properties to help us perform further analyses. In the vicinity of the scatterer, under the high-*Q* approximation where the FF part of the wave function is significantly weaker than the NF part, $\psi _m^{{\mathrm{NF}}}$ can be approximately viewed as the eigenfunction of the Hamiltonian $\widehat {\mathbf{{{H}}}}_m$ with a real eigenvalue *ω*_*m*_,

3 $$\widehat {\mathbf{{{H}}}}_m\left| {\psi _m^{{\mathrm{NF}}}} \right\rangle \approx \omega _m\left| {\psi _m^{{\mathrm{NF}}}} \right\rangle $$

Meanwhile, $\psi _m^{{\mathrm{NF}}}$ can be normalized since it is well localized around the *m*th resonator. Moreover, considering that these wave functions are spatially well separated, we find that they approximately satisfy the following orthonormal condition:

4 $$\left\langle {{\psi _m^{{\mathrm{NF}}}}} | {{\psi _n^{{\mathrm{NF}}}}} \right\rangle _V\, \approx\, \delta _{mn}$$

where the integrals are performed over the entire space. We note that one needs to multiply $\psi _m^{{\mathrm{LEM}}}$ by the same normalization constant that is used to normalize $\psi _m^{{\mathrm{NF}}}$, since these two functions are connected by Eq. (2). Equation (4) indicates that $\{ \psi _m^{{\mathrm{NF}}},m = 1,...,M\}$ form a set of orthogonal bases to expand the total wave functions in the NF region. Note that the approximation Eq. (4) is widely used in the TBM for treating electrons in solids^26^.

We now identify the FF eigenbases of the system. In the FF region, eigenmodes are just a set of propagating modes $\left\{ {\left| {k_q^ \pm } \right\rangle } \right\}$ allowed by the system, where +(−) denotes the incoming (outgoing) propagation direction, *q* labels the mode channel, and *k* is the wavevector satisfying certain dispersion relation *k*_*q*_(*ω*). These wave functions satisfy the following orthogonal condition:

5 $$\left\langle {{k_p^\sigma }} | {{k_q^{\sigma^\prime }}} \right\rangle _S = \delta _{pq}^{\sigma \sigma^\prime }$$

where the integrals are performed on the reference plane of a particular external port. In principle, extending our theory to study cases with continuum scattering ports^36,37^ is also possible, although one needs to compute all parameters related to these scattering channels.

We are now ready to represent Ψ as a linear combination of these basis functions. We have Ψ = Ψ_B_ + Ψ^sca^, where Ψ^sca^ is contributed by the scatterings of all resonators. In the same spirit as the TBM, Ψ^sca^ can be approximately written as a sum of scattered fields ${\Psi} _{^m}^{{\mathrm{sca}}}$ associated with each individual scatterer. At first glance, one may expect that ${\Psi} _{^m}^{{\mathrm{sca}}}(\vec r,\omega )$ must be $\psi _{^m}^{{\mathrm{LEM}}}(\vec r,\omega _m)$ defined previously. However, $\psi _{^m}^{{\mathrm{LEM}}}(\vec r,\omega _m)$ is the scattered wave at resonance frequency *ω*_*m*_, not at arbitrary frequencies as required in Eq. (1). We can amend $\psi _{^m}^{{\mathrm{LEM}}}(\vec r,\omega _m)$ slightly to obtain the form of $\psi _{^m}^{{\mathrm{LEM}}}(\vec r,\omega )$ for a frequency *ω* not far from *ω*_*m*_. The NF part [$\psi _m^{{\mathrm{NF}}}(\vec r,\omega _m)$] is solely determined by *ω*_*m*_, as it is (approximately) an eigensolution of Eq. (3) for eigenfrequency *ω*_*m*_. Since, we will need to utilize the orthonormal properties of $\psi _m^{{\mathrm{NF}}}(\vec r)$ offered by Eq. (3) later, here, we take the original form of $\psi _m^{{\mathrm{NF}}}(\vec r)$ in constructing the trial wave functions at general frequencies *ω* ≠ *ω*_*m*_. Meanwhile, the FF part $\psi _m^{{\mathrm{FF}}}(\vec r,\omega _m)$ contains propagating terms depending on the wavevector *k*_*q*_, which must be modified from *k*_*q*_(*ω*_*m*_) to *k*_*q*_(*ω*) according to the dispersion relations. We note, however, that $\psi _m^{{\mathrm{LEM}}}(\vec r,\omega )$ thus obtained neglects the frequency corrections to the FF radiation amplitudes. In principle, such corrections can be taken into account by considering the NF–FF relation of a given source^38,39^. To obtain a concise analytical form for our theory, here, we neglected such corrections, justified by the high-*Q* approximation. Later, we show that such an approximation works quite well even though the original modes supported by individual resonators do not exhibit extremely high-*Q* factors.

We can finally construct the total wave function as

6 $${\Psi} (\vec r,\omega ) = \mathop {\sum}\limits_q {s_q^ + {\Psi} _{\mathrm{B}}^q} {\mathrm{ + }}\mathop {\sum}\limits_n {a_n} \left| {\psi _n^{{\mathrm{LEM}}}} \right\rangle $$

where {*a*_*n*_} are a set of unknown coefficients representing the strengths of fields scattered by different resonators under external illumination represented by $\{ s_q^ + \}$ denoting the excitation amplitudes at different incoming ports, and ${\Psi} _{\mathrm{B}}^q$ denotes the background wave function obtained when only the *q*th port is excited with unit amplitude. Substituting Eq. (6) into Eq. (1), projecting both sides by$\left\langle {\psi _m^{{\mathrm{NF}}}} \right|$ and utilizing the orthogonal condition Eq. (4), we obtain the following equations to determine{*a*_*n*_}:

7 $$- i\omega a_m = - i(\omega _m - i{\Gamma} _m)a_m + {\sum \limits_{n \ne m}} {( - it_{mn} + X_{mn})} a_n +{\sum \limits_q} {\kappa _{mq}s_q^ + } $$

We next multiply both sides of ${\Psi} (\vec r,\omega )$ defined in Eq. (6) by each FF outgoing basis $\left\langle {k_q^ - } \right|$, and then perform the field integrations at the reference planes of all ports. Using the orthonormal conditions Eq. (5), we finally obtain the set of equations:

8 $$s_q^ - = \mathop {\sum}\limits_p {s_p^ + } c_{qp} + \mathop {\sum}\limits_m {a_m} d_{qm}$$

to determine $\left\{ {s_q^ - = \left\langle {{k_q^ - }} | {{\Psi} } \right\rangle _S} \right\}$, which describe the strengths of scattered fields measured at different external ports. Here, all parameters in Eqs. (7) and (8) are unambiguously defined and can be calculated via the following integrals:

9 $$\left\{ {\begin{array}{*{20}{c}} {{\Gamma} _m = i\left\langle {\psi _m^{{\mathrm{NF}}}} \right|\widehat {\mathbf{V}}_m\left| {\psi _m^{{\mathrm{FF}}}} \right\rangle _V} \\ {t_{mn}{\mathrm{ = }}\left\langle {\psi _m^{{\mathrm{NF}}}} \right|\widehat {\mathbf{V}}_m\left| {\psi _n^{{\mathrm{NF}}}} \right\rangle _V} \\ {X_{mn} = - i\left\langle {\psi _m^{{\mathrm{NF}}}} \right|\widehat {\mathbf{V}}_m\left| {\psi _n^{{\mathrm{FF}}}} \right\rangle _V} \\ {\kappa _{mq}{\mathrm{ = }} - i\left\langle {\psi _m^{{\mathrm{NF}}}} \right|\widehat {\mathbf{V}}_m\left| {{\Psi} _{\mathrm{B}}^q} \right\rangle _V} \\ {c_{qp} = \left\langle {{k_q^ - }} | {{{\Psi} _{\mathrm{B}}^q}} \right\rangle _S} \\ {d_{qm} = \left\langle {{k_q^ - }} | {{\psi _m^{{\mathrm{FF}}}}} \right\rangle _S} \end{array}} \right.$$

where “*V*” and “*S*” denote whether the integrals are performed over the entire volume or at the reference plane of a port. The physical meanings of all involved parameters can be clearly seen from their expressions. For example, *t*_*mn*_ and *X*_*mn*_ represent the coupling strengths between two resonators due to their NF and FF interactions, respectively. Derivations of Eqs. (7)–(9) can be found in Sec. II of the Supplementary Information.

It is helpful to explicitly discuss the conditions imposed on our systems to make the derived theory (e.g., Eqs. (7)–(9)) valid. By re-examining Eq. (7) for the single-scatterer case, we find that Im(Γ_*m*_), if it exists, can shift the resonance frequency *ω*_*m*_, and thus, a large Im(Γ_*m*_) implies that $\psi _m^{{\mathrm{NF}}}$ is not reasonably chosen. Therefore, the first criterion is ${\rm{Im}}({\Gamma} _m) \to 0$, which determines the accuracy of our theory at resonance. Meanwhile, we also require ${\rm{Re}}({\Gamma} _m) \,< <\, \omega _m$, which is responsible for the correctness of our theory in describing the entire lineshape. The second criterion can be easily satisfied by a moderate *Q* value (e.g., *Q* > 5), as long as the frequency dispersion of the material is not significant and high-order modes are all far from the mode under study. The first criterion, however, requires the resonators to be deep subwavelength in size so that $\psi _m^{{\mathrm{FF}}}$ and $\psi _m^{{\mathrm{NF}}}$ can exhibit a *π*/2 phase difference inside the whole region occupied by the resonator^38^, leading to a negligible Im(Γ_*m*_). For plasmonic resonances, such a deep-subwavelength condition is easily satisfied. However, for dielectric resonances, such a condition can only be satisfied in systems with a very high refraction index (*n*), which pushes the *Q* factors to even higher values (see Sec. IX in the Supplementary Information for more details).

We note that Eq. (9) is derived for lossless systems, and thus, Γ_*m*_ must only contain radiation damping. In realistic systems, we also need to consider another parameter ${\Gamma} _m^{\mathrm{a}}$, representing the damping due to absorption (i.e., replacing Γ_*m*_ by ${\Gamma} _m + {\Gamma} _m^{\mathrm{a}}$ in Eq. (8)). This parameter can be computed using ${\Gamma} _m^{\mathrm{a}}=i\left\langle {\psi _m^{{\mathrm{NF}}}} \right|(\widehat {\mathbf{{{H}}}}_m^{\mathrm{a}} - \widehat {\mathbf{{{H}}}}_m^0)\left| {\psi _m^{{\mathrm{NF}}}} \right\rangle _V$, where $\widehat {\mathbf{{{H}}}}_m^{\mathrm{a}}$ represents the Hamiltonian of the realistic lossy systems, while $\widehat {\mathbf{{{H}}}}_m^0$ describes the same system with material losses omitted^40^.

Equations (7)–(9) are the core results of this paper, which have clear and profound physical meanings. While Eq. (7) describes the dynamics of each mode under certain excitations, Eq. (8) describes the measurable scattering spectra. We note that Eqs. (7) and (8) resemble the two equations in CMT^18,19^, but our theory is different and possesses the following merits. In the empirical CMT, the key parameters defined are usually obtained by fitting with numerical simulations, while the remaining parameters can be derived by energy-conservation and time-reversal arguments^41^. In contrast, here, in our theory, all parameters can be unambiguously evaluated by Eq. (9), and therefore, one can use it to predict the lineshapes of coupled systems before performing numerical simulations on them. Moreover, the empirical CMT cannot explicitly consider the NF couplings between resonators^18^, while in our approach, NF couplings *t*_*mn*_ can be unambiguously determined (see Eq. (9)) and explicitly included in determining the lineshape (Eq. (8)). Although single-resonator parameters (*ω*_res_ and Γ_res_) can be analytically obtained for certain high-symmetry structures for that analytical formulas of scattering coefficients are available^42^, such an approach is not general enough to deal with arbitrary coupled systems without analytical expressions of scattering coefficients and cannot be used to study the couplings between different resonators.

### Applications to photonic systems and benchmark tests

We now apply the developed formal theory to photonic systems, described generally by an inhomogeneous permittivity function $\varepsilon (\vec r,\omega )$, in which at each local point $\vec r$, the permittivity is $\varepsilon (\omega ) = \varepsilon _\infty [1 + \omega _p^2/(\omega _0^2 - \omega ^2 + i\omega {\Gamma} _e)]$, where *ε*_∞_, *ω*_0_, *ω*_*p*_, and Γ_*e*_ are all position- and frequency-independent parameters, describing the local properties of constituent materials. The governing equations (i.e., Maxwell’s equations in the frequency domain) can be formally rewritten as Eq. (1)^40^, where the Hamiltonian is given by

10 $$\widehat {\mathbf{{{H}}}} = \left( {\begin{array}{*{20}{c}} 0 & { - \frac{i}{\mu }\nabla \times } & 0 & 0 \\ {\frac{i}{{\varepsilon _\infty }}\nabla \times } & 0 & 0 & { - \frac{i}{{\varepsilon _\infty }}} \\ 0 & 0 & 0 & i \\ 0 & {i\omega _p^2\varepsilon _\infty } & { - i\omega _0^2} & { - i{\Gamma} _e} \end{array}} \right)$$

and the wave function is defined as $${\Psi} (\vec r) = \left( {\begin{array}{*{20}{c}} {\vec H} & {\vec E} & {\vec P} & {\vec V} \end{array}} \right)^T$$, with $\vec E$, $\vec H$, and $\vec P$ denoting the electric, magnetic, and polarization fields, respectively, and $\vec V = {\mathrm{d}}\vec P/{\mathrm{d}}t$ describing the polarization current. Consider the lossless case first (i.e., Γ_*e*_ = 0). The inner product between two wave functions is defined as^26,40^

11 $$\begin{array}{l}\left\langle {\psi _1(\vec r)|\psi _2(\vec r)} \right\rangle _V\\ = \frac{1}{2}{\int} {{\mathrm{d}}\tau [\mu \vec H_1^ \ast \cdot \vec H_2 + \varepsilon _\infty \vec E_1^ \ast \cdot \vec E_2 + \omega _0^2(\omega _p^2\varepsilon _\infty )^{ - 1}\vec P_1^ \ast \cdot \vec P_2 + (\omega _p^2\varepsilon _\infty )^{ - 1}\vec V_1^ \ast \cdot \vec V_2]} \end{array}$$

Meanwhile, in the FF region occupied by air, the inner product between two-port modes can be defined as

12 $$\left\langle {k_1^\sigma |k_2^{\sigma \prime }} \right\rangle _S = \frac{1}{2}{\oint} {[\mu _0(\vec H_1^\sigma )^ \ast \cdot \vec H_2^{\sigma^{\prime}} + \varepsilon _h(\vec E_1^\sigma )^ \ast \cdot \vec E_2^{\sigma^{\prime}}]\vec c \cdot {\mathrm{d}}\vec S} $$

where $\vec c$ is the light speed in the host medium. This ensures that different port modes are orthogonal and that each mode carries a unit of energy flux^43^. With Eqs. (10)–(12) and supposing that $\{ \psi _m^{{\mathrm{NF}}},\psi _m^{{\mathrm{FF}}}\}$ are obtained, one can substitute them into Eq. (9) to compute all parameters (see Sec. III in the Supplementary Information) and then substitute them into Eqs. (7) and (8) to determine the lineshape.

For photonic resonators with regular shapes, $\{ \psi _m^{{\mathrm{NF}}},\psi _m^{{\mathrm{FF}}}\}$ can be obtained analytically. For arbitrary resonators, we need to numerically obtain the required wave functions. We emphasize that, however, such numerical calculations are only needed once. Once $\{ \psi _m^{{\mathrm{NF}}},\psi _m^{{\mathrm{FF}}}\}$ are obtained, we can predict the lineshapes of the coupled systems without having to perform simulations on them.

**Fig. 2:**
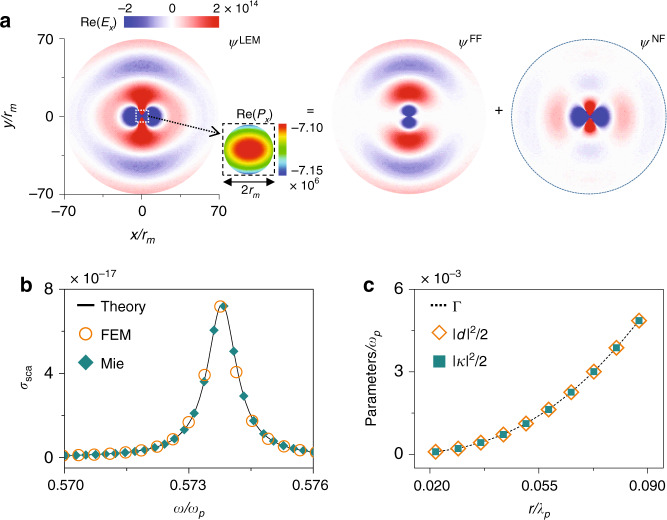
Benchmark test against Mie theory. **a** Distributions of the *E*_*x*_-component in the *ψ*^LEM^, *ψ*^FF^, and *ψ*^NF^ of a gold sphere (with *r*_*m*_ = 0.036*λ*_*p*_) obtained by Mie theory. The inset shows the distribution of *P*_*x*_ inside the sphere at the central *xoy* plane. **b** Spectra of the scattering cross section of the gold sphere derived by our theory (line), FEM calculations (circles), and the Mie solution (squares). **c** *κ*, *d*, and Γ of a series of gold spheres with different sizes, computed by our theory

We first choose an analytically solvable system—a single gold sphere illuminated by an *x*-polarized plane wave—to test our theory against Mie theory. As shown in Fig. 2a, consider a sphere located at the origin with radius *r*_*m*_ = 0.036*λ*_*p*_ and Drude permittivity $\varepsilon (\omega ) = \varepsilon _0[1 - \omega _p^2{\mathrm{/}}\omega ^2]$, with *ω*_*p*_ and *λ*_*p*_ denoting the plasmon resonance frequency and wavelength. Such a problem can be analytically solved by Mie theory^44,45^, yielding an analytical form of ${\Psi} ^{{\mathrm{sca}}}(\vec r,\omega )$. When the scatterer is much smaller than the wavelength of incident light, the electric dipole channel dominates in the frequency range plotted^46^, and thus, we can obtain $\omega _{{\mathrm{res}}} = [1 - 8\pi ^2(r_m/\lambda _p)^2/15 + ...]\omega _p/\sqrt 3$ and the analytical forms of $\psi^{\rm{LEM}},\psi^{\rm{FF}}$, and $\psi^{\rm{NF}}$, as well as $\left| {k^ \pm } \right\rangle$ (see Sec. IV in the Supplementary Information). Figure 2a depicts the field distributions of $\psi^{\rm{LEM}},\psi^{\rm{FF}}$, and $\psi^{\rm{NF}}\, {.} \,\psi^{\rm{NF}}$ exhibits a clear electric dipole resonance feature, and $\psi^{\rm{FF}}$ represents the FF radiation of an electric dipole located at the origin.

Substituting all wave functions into Eq. (9), we find $\kappa = d = 2.92 \times 10^{ - 2}\sqrt {\omega _p} i$ and ${\Gamma} = 4.28 \times 10^{ - 4}\omega _p$. Since there is only one scatterer and one port in the system, we neglect all subscripts without causing confusion. Substituting these parameters into Eqs. (7) and (8), we obtain the scattering spectrum of the nanosphere, defined as$\sigma (\omega ) = 3\pi \left| {1 - R} \right|^2/(2\eta _0k_0^2)$, with $\eta _0 = \sqrt {\mu _0/\varepsilon _0}$ being the vacuum impedance and $R \equiv s_{}^ - /s_{}^ +$, representing the scattering coefficient. The spectrum thus calculated is depicted in Fig. 2b as a solid line, well matching the Mie theory (squares) and FEM calculation (circles) results.

Under the electric dipole approximation, we further simplify the analytical expressions of all involved parameters (see Sec. IV in the Supplementary Information) as

13 $$\left\{ {\begin{array}{*{20}{c}} {{\Gamma} = \omega _{{\mathrm{res}}} \times \rm{Im}\, \{ \vec p^ \ast \times \vec E^{{\mathrm{FF}}}\} {\mathrm{/}}2 = p^2\omega _{{\mathrm{res}}}^{\mathrm{4}}{\mathrm{/}}\left( {12\pi \varepsilon _0c_0^3} \right)} \\ {\kappa = ip\omega _{{\mathrm{res}}}^{\mathrm{2}}{\mathrm{/}}\sqrt {6\pi \varepsilon _0c_0^3} } \\ {d = ip\omega _{{\mathrm{res}}}^{\mathrm{2}}{\mathrm{/}}\sqrt {6\pi \varepsilon _0c_0^3} } \end{array}} \right.$$

with $\vec p_{} = {\int}_{{\mathrm{sphere}}} {\vec P_{}(\vec r){\mathrm{d}}\vec r}$ ($\vec P$ is the polarization field inside the sphere; see the inset in Fig. 2a), representing the effective dipole moment of the nanosphere. Equation (13) reveals a few important physics difficult to obtain from numerical calculations. First, *κ* and *d*, defined as two distinct field integrations (Eq. (9)), surprisingly generate identical results (see Eq. (13)), which is consistent with the time-reversal symmetry argument^19^. Second, Γ takes an expression identical to that derived for a dipole emitter based on Poynting’s theorem (see Eq. (8.74) in ref. ^38^), revealing the clear physical meaning of the radiation damping. Finally, Eq. (13) uncovers the relation $2{\Gamma} = \left| p \right|^2\omega _{{\mathrm{res}}}^{\mathrm{4}}/(6\pi \varepsilon _0c^3) = \left| d \right|^2$ verified by numerical calculations (see Fig. 2c), which ensures energy conservation consistent with Poynting’s theorem^38^. We note that these relations were derived by energy-conservation and time-reversal arguments in the empirical CMT. Here, they are directly and rigorously demonstrated in our theory simply because our theory is established based on Maxwell’s equations, which already satisfy energy-conservation and time-reversal symmetry.

**Fig. 3:**
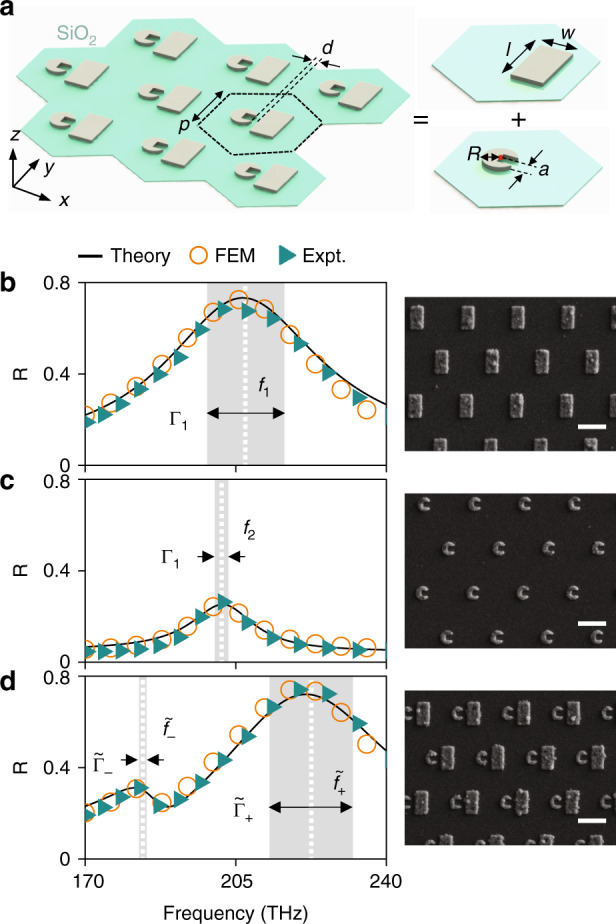
Benchmark test of our theory on a realistic system. **a** Schematic of the coupled plasmonic system under study. Here, the geometrical parameters are *p* = 530, *d* = 30, *w* = 240, *l* = 420, *R* = 110, and *a* = 85, all in units of nm. **b**–**d** Reflectance spectra of periodic metasurfaces containing **b** bar resonators only, **c** C resonators only, and **d** the two resonators coupled together, obtained by our theory (solid lines), FEM simulations (circles), and measurements (triangles). White dashed lines and gray areas denote the frequencies and widths of the resonant modes. The right panels of **c** and **d** are SEM images of the fabricated samples with scale bars (white lines) of 500 nm

After studying coupled electric dipole resonators to justify our theory against analytical formulas derived in prior literature^47,48^ (see Sec. V in the Supplementary Information for details), we implement our theory to study arbitrary photonic coupled systems. As shown in Fig. 3a, the system we consider is a periodic metasurface with unit cells arranged in a hexagonal lattice (with periodicity 550 nm), each containing two different types of nanoparticles (bar and C-shaped resonator) coupled together. All nanoparticles are made of silver and are placed on a semi-infinite dielectric substrate (*n* = 1.55). Following the general strategy established above, we first perform lossless FEM simulations to study the scattering properties of two model systems, each containing resonators of a particular type arranged in the same hexagonal lattice (see Fig. 3a). Due to the periodic arrangements with deep-subwavelength spacing, only the zero-order transmission/reflection channels survive in the FF. From the calculated reflection spectra (circles) shown in Fig. 3b, c, we identify the resonance frequencies {*ω*_*m*_, *m* = 1, 2} of the two resonators (see dashed lines in Fig. 3b, c). We then follow the general strategy described in the last section to determine the needed NF and FF wave functions $\{ \psi _m^{{\mathrm{FF}}},\psi _m^{{\mathrm{NF}}},m = 1,2\}$. Substituting these single-resonator properties into Eq. (9), we obtain all needed parameters (see Sec. VI in the Supplementary Information for details) and, in turn, the desired transmission/reflection spectra. The reflectance spectra calculated by our theory are plotted in Fig. 3b, c as black lines, in perfect agreement with FEM simulations (circles) of realistic structures. This is remarkable since we did not perform any fitting procedures in obtaining these spectra. The lineshape of the coupled system predicted by our theory is further confirmed by our experiments. We fabricated three samples according to the designs using the standard electron-beam lithography (EBL) method (see left panel in Fig. 3b–d for their scanning electron microscopy (SEM) images) and experimentally characterized their reflection spectra. The spectra of the three samples are shown in Fig. 3b–d as triangles, measured with a homemade macroscopic spectrometer (see Sec. VII in the Supplementary Information). The excellent agreement among the FEM, the experimental and our theoretical results unambiguously justify our theory.

### Implementations of the theory in lineshape tailoring

We now apply our theory to “design” the lineshape of a photonic system. Figure 3d shows that the interresonator coupling can dramatically change the lineshape of a coupled system, essentially determined by the two “dressed” modes with frequencies and bandwidths $\{ \tilde \omega _ \pm ,\tilde {\Gamma} _ \pm \}$. Therefore, we must first understand the properties of the dressed modes $\{ \tilde \omega _ \pm ,\tilde {\Gamma} _ \pm \}$.

Consider a two-mode two-port system with two resonators placed on the same plane illuminated by a normally incident wave. Assuming ${\Gamma} _{\mathrm{1}}^{\mathrm{a}} = {\Gamma} _2^{\mathrm{a}} = {\Gamma} ^{\mathrm{a}}$ for simplicity, we can explicitly rewrite Eq. (7) as

14 $$- i\omega \left( {\begin{array}{*{20}{c}} {a_1} \\ {a_2} \end{array}} \right) = - i\left[ {\left( {\begin{array}{*{20}{c}} {\omega _1} & t \\ t & {\omega _2} \end{array}} \right) - i\left( {\begin{array}{*{20}{c}} {{\Gamma} ^{\mathrm{a}}} & 0 \\ 0 & {{\Gamma} ^{\mathrm{a}}} \end{array}} \right)} \right]\left( {\begin{array}{*{20}{c}} {a_1} \\ {a_2} \end{array}} \right) + \left( {\begin{array}{*{20}{c}} { - {\Gamma} _1} & X \\ X & { - {\Gamma} _2} \end{array}} \right)\left( {\begin{array}{*{20}{c}} {a_1} \\ {a_2} \end{array}} \right) + \left( {\begin{array}{*{20}{c}} {\kappa _{11}} \\ {\kappa _{21}} \end{array}} \right)s_1^ + $$

Diagonalizing the matrix containing *t* by an orthogonal transformation **M**, we obtain the following equation describing the amplitudes of two collective modes $\tilde a_ \pm$:

15 $$- i\omega \left( {\begin{array}{*{20}{c}} {\tilde a_ + } \\ {\tilde a_ - } \end{array}} \right) = - i\left[ {\left( {\begin{array}{*{20}{c}} {\tilde \omega _{\mathrm{ + }}} & 0 \\ 0 & {\tilde \omega _ - } \end{array}} \right) - i\left( {\begin{array}{*{20}{c}} {\tilde {\Gamma} ^{\mathrm{a}}} & 0 \\ 0 & {\tilde {\Gamma} ^{\mathrm{a}}} \end{array}} \right)} \right]\left( {\begin{array}{*{20}{c}} {\tilde a_ + } \\ {\tilde a_ - } \end{array}} \right) + \left( {\begin{array}{*{20}{c}} { - \tilde {\Gamma} _ + } & {\tilde X} \\ {\tilde X} & { - \tilde {\Gamma} _ - } \end{array}} \right)\left( {\begin{array}{*{20}{c}} {\tilde a_ + } \\ {\tilde a_ - } \end{array}} \right) + \left( {\begin{array}{*{20}{c}} {\tilde \kappa _{11}} \\ {\tilde \kappa _{21}} \end{array}} \right)s_1^ + $$

where $\tilde {\Gamma} _ \pm = ({\Gamma} _1 + {\Gamma} _2)/2 \pm (2t\sqrt {{\Gamma} _1{\Gamma} _2} + {\Delta} \omega {\Delta} {\Gamma} )/(2\sqrt {t^2 + {\Delta} \omega ^2} )$, $\tilde \omega _ \pm = (\omega _1 + \omega _2)/2 \pm \sqrt {t^2 + {\Delta} \omega ^2}$, with ${\Delta} \omega = (\omega _1 - \omega _2)/2$ and ${\Delta} {\Gamma} = {\Gamma} _1 - {\Gamma} _2$, and $$\left( {\begin{array}{*{20}{c}} {\tilde a_ + } & {\tilde a_ - } \end{array}} \right)^{\mathrm{T}} = {\mathbf{M}}\left( {\begin{array}{*{20}{c}} {a_1} & {a_2} \end{array}} \right)^{\mathrm{T}}$$. Since an orthogonal transformation does not change the trace of a matrix, it is sufficient to study ${\Delta} \tilde \omega = \tilde \omega _ + - \tilde \omega _ -$ and ${\Delta} \tilde {\Gamma} = \tilde {\Gamma} _ + - \tilde {\Gamma} _ -$, which are determined by *t*, Δ*ω*, and ΔΓ via

16 $$\left\{ {\begin{array}{*{20}{c}} {{\Delta} \tilde \omega = 2\sqrt {t^2 + ({\Delta} \omega )^2} } \\ {{\Delta} \tilde {\Gamma} = (2t + {\Delta} \omega \times {\Delta} {\Gamma} )/\sqrt {t^2 + ({\Delta} \omega )^2} } \end{array}} \right.$$

Here, and in what follows, we have scaled all involved physical quantities (i.e., ${\Delta} \tilde \omega$, ${\Delta} \tilde {\Gamma}$, Δ*ω*, ΔΓ, and *t*) by $\sqrt {{\Gamma} _1{\Gamma} _2}$ to make them dimensionless. Equation (16) shows that even for two resonators with fixed properties, one can still use the interresonator coupling *t* to change the properties of the “dressed” modes and, in turn, “design” the final lineshape of the coupled system.

**Fig. 4:**
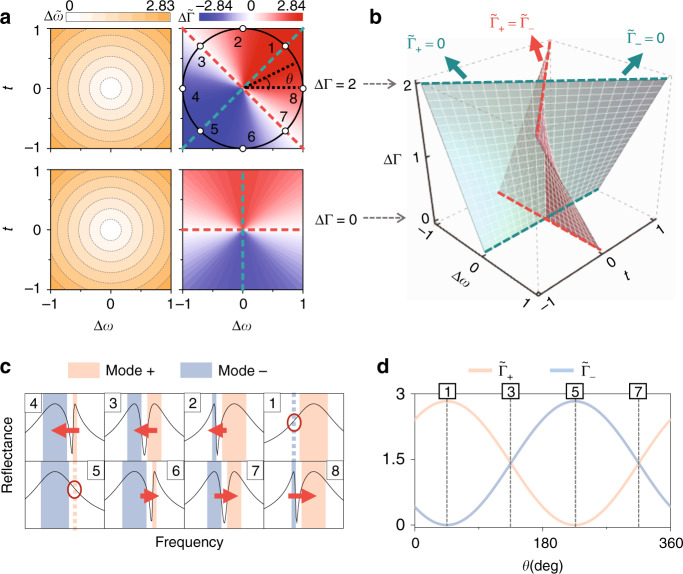
Phase diagram for lineshape tailoring. **a** Frequency difference ${\Delta} \tilde \omega$ (left panels) and radiation-damping difference ${\Delta} \tilde {\Gamma}$ (right panels) of the “dressed” modes versus Δ*ω* and *t*, with ΔΓ fixed at 2 (upper panels) and 0 (bottom panels), for a two-mode two-port system. **b** Phase-separation surfaces defined by ${\Delta} \tilde {\Gamma} = 0$ (red surface) and ${\Delta} \tilde {\Gamma} = \pm ({\Gamma} _1 + {\Gamma} _2)$ (green surface) versus Δ*ω*, *t*, and ΔΓ. **c** Optical lineshapes of the systems corresponding to the eight points defined on the black circle in the upper right panel of **a**. **d** Evolutions of the radiation damping of the two dressed modes ($\tilde {\Gamma} _{\mathrm{ + }}$ and $\tilde {\Gamma} _ -$) while moving on the circle defined in the upper right panel of **a**. Here, all quantities are scaled by $\sqrt {{\Gamma} _1{\Gamma} _2}$

The left and right panels in Fig. 4a depict, respectively, how ${\Delta} \tilde \omega$ and ${\Delta} \tilde {\Gamma}$ vary with Δ*ω* and *t*, with ΔΓ set at two different values. We find that while ${\Delta} \tilde \omega$ exhibits circular equal-value lines on the Δ*ω* ~ *t* plane independent of ΔΓ, ${\Delta} \tilde {\Gamma}$, exhibits fascinating behavior on the Δ*ω* ~ *t* plane depending sensitively on ΔΓ. In particular, on each Δ*ω* ~ *t* phase plane with a fixed ΔΓ, we always find two special lines, defined as ${\Delta} \tilde {\Gamma} = 0$ (red lines) and ${\Delta} \tilde {\Gamma} = \pm ({\Gamma} _1 + {\Gamma} _2)$ (green lines), to separate the whole space into four subregions with distinct properties. Physically, while the condition ${\Delta} \tilde {\Gamma} = 0$ implies that the two dressed modes have identical bandwidths (i.e., $\tilde {\Gamma} _{\mathrm{ + }} = \tilde {\Gamma} _ -$), the other condition ${\Delta} \tilde {\Gamma} = \pm ({\Gamma} _1 + {\Gamma} _2)$ means that one dressed mode exhibits vanishing radiative damping. Interestingly, these two phase boundary lines rotate as ΔΓ changes, as shown in Fig. 4b.

To illustrate the key features of the four subregions, we purposely choose eight points from a circle on the Δ*ω* ~ *t* plane with ΔΓ = 2 (see Fig. 4a) and illustrate in Fig. 4c how the reflection spectra of the corresponding systems evolve. Consistent with our expectations, the spectra of systems 1 and 5 only exhibit one peak, as the other mode is completely dark, while the spectra of systems 3 and 7 exhibit two peaks with equal bandwidths. In between these special points, the spectra gradually evolve. Notably, the radiation damping (bandwidths) of the two “dressed” modes can vary continuously from 0 to Γ_1_ + Γ_2_, while moving on the circle (see Fig. 4d).

The physics is very clear: now that the dressed modes are appropriate linear combinations of two original modes, their radiation damping must also be linear combinations of that of the two original modes. Therefore, varying Δ*ω* and *t* can dramatically modify the relative portions of the two original modes in constructing the dressed modes and, in turn, efficiently control the radiation damping of the dressed modes. In principle, one can realize any desired lineshapes based on our phase diagram by choosing certain original modes and “tuning” the coupling *t*. Of particular interest is the appearance of a purely dark mode with infinitely long lifetime, which shares the same physical origin as the BIC and has many interesting applications^49–51^.

**Fig. 5:**
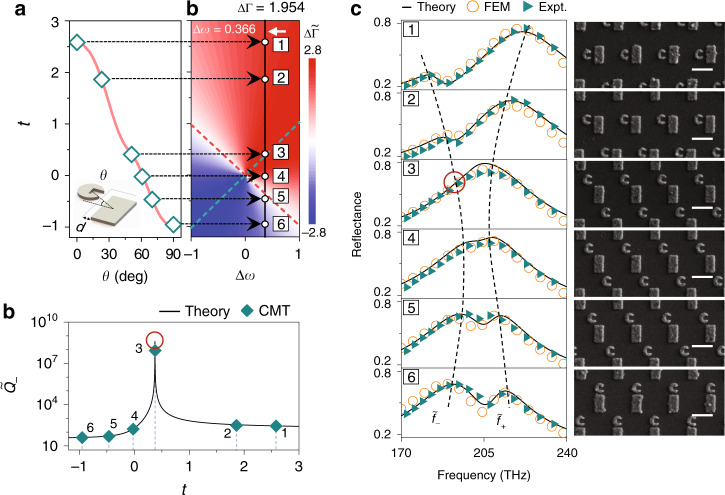
Experimental demonstrations of lineshape tailoring. **a** Calculated interresonator coupling strength *t* as a function of the relative angle *θ* between two resonators with *d* fixed at 30 nm (see inset). Open squares represent six chosen systems experimentally tested. **b** Positions of the six chosen systems on the Δ*ω* ~ *t* phase diagram. The two original modes have fixed ΔΓ = 1.954 and Δ*ω* = 0.733. **c** Reflectance spectra of six systems obtained by our theory (lines), FEM simulations (open circles), and experimental measurements (solid triangles) on realistic samples, with SEM images shown in the right panels with scale bars (white lines) of 500 nm. The two dashed lines indicate the frequencies of the two dressed modes, and the red circle on the spectrum of sample 3 denotes the BIC. **d** Quality factor of the low-frequency dressed mode $\tilde Q_ -$ as a function of the interresonator coupling *t* obtained by our theory (line), and retrieved from experimental spectra by fitting with CMT (rhombus*)*. Δ*ω*, ΔΓ, and *t* are all scaled by $\sqrt {{\Gamma} _1{\Gamma} _2}$

We now experimentally verify our predictions on lineshape tailoring based on coupled systems constructed by the two resonators studied in Fig. 3a. Since *t* is solely determined by the overlap between the $\psi _m^{{\mathrm{NF}}}$ of two resonators (see Eq. (9)), we understand that changing the resonators’ relative configuration can dramatically modify *t*. Indeed, as we rotate the C-shaped resonator with respect to the bar resonator, we find that *t* drastically changes (see solid line in Fig. 5a). In particular, increasing the relative angle *θ* between two resonators can drive *t* to change from a positive value to a negative value, passing through 0 at a particular angle. Such an intriguing *t* ~ *θ* relation can be simply explained by an effective model for plasmonic coupling established previously^47,48^.

Choosing six points on the *t* ~ *θ* curve, as shown in Fig. 5a, we employ our theory to study the optical lineshapes of their corresponding realistic systems. Since the two original modes have fixed properties, these six systems with different *t* are located on a straight line in the phase diagram passing through two phase boundaries (see Fig. 5b). Their reflection spectra, computed by our theory, are depicted in Fig. 5c as solid lines, exhibiting the expected behaviors. In particular, the spectrum of the third system only exhibits one peak, while that of the fifth system contains two equal-bandwidth peaks, consistent with the phase diagram shown in Fig. 5b. Once again, we emphasize that all spectra are calculated with our theory directly and without any fitting procedures.

We then perform both experiments and simulations to verify the above theoretical predictions. We fabricate samples according to the designs using the standard EBL method, with the right panel in Fig. 5c showing SEM images of the fabricated samples. Illuminating these samples with normally incident light with $\vec E\parallel \hat y$, we measure their transmission/reflection spectra and depict the reflection spectra as solid triangles in Fig. 5c. We also perform FEM simulations to calculate their reflection spectra (open circles in Fig. 5c). Both the experimental and simulation results are in excellent agreement with the spectra obtained by our theory (solid lines in Fig. 5c). In particular, the measured/simulated spectra of sample 3 exhibit clear BIC features, while those of sample 5 contain two peaks with equal bandwidths. We also employ our theory to predict the transmission spectra of these systems, which are in excellent agreement with the measured and simulated results (see Sec. VIII in the Supplementary Information).

The solid line in Fig. 5d depicts how varying *t* significantly modulates the radiative *Q* factor of the low-frequency dressed mode, as predicted by our theory. That the *Q* factor diverges at a specific point signifies the appearance of a BIC. The symbols are the *Q* factors of six realistic samples obtained by analyzing their measured reflection spectra. Excellent agreement is noted between the experimental and analytical results. At the frequency where the BIC appears, the radiations from the two individual resonators exactly cancel each other, leading to vanishing of the total radiative loss (see Sec. VI in the Supplementary Information).

## Discussion

In summary, we have derived a formal theoretical framework directly from Maxwell’s equations to study the optical responses of arbitrarily coupled photonic systems, in which all involved parameters are unambiguously computable without any fitting procedures. After testing it against both Mie theory and numerical simulations on different systems, we illustrate how to employ it to design the lineshape of a coupled system by modulating the couplings between resonators. In particular, we show that one can always choose a specific coupling between two arbitrary resonators to make one of the “dressed” modes in the coupled system completely dark, creating a BIC. All predictions are quantitatively verified by our experiments and simulations at near-infrared wavelengths. In addition to revealing the profound physics underlying the coupling-induced phenomena, our theory also offers a powerful tool to design optical devices with well-controlled NF and FF properties, and can be extended to study coupled systems for other types of waves.

## Materials and methods

### Simulations

We employed FEM simulations using the commercial software COMSOL Multiphysics. The permittivity of Ag was described by the Drude model $\varepsilon (\omega ) = \varepsilon _\infty - \frac{{\omega _p^2}}{{\omega (\omega \,+\, i{\mathrm{{\Gamma} }}_e)}}$, with $\varepsilon _\infty = 5\varepsilon _0$, $\omega _0 = 0\,{\mathrm{THz}}$, and $\omega _p = 2\pi \times 2176.2\,{\mathrm{THz}}$. The effective damping rate was set as ${\Gamma} _e = 2\pi \times 38.3\,{\mathrm{THz}}$ for the bar structure and ${\Gamma} _e = 2\pi \times 27.3\,{\mathrm{THz}}$ for the C-shaped resonator, obtained by fitting with our experimental results. The SiO_2_ spacer was considered a lossless dielectric with permittivity *ε* = 2.42. Additional losses caused by surface roughness and grain boundary effects in thin films, as well as dielectric losses were effectively considered in the fitting parameter Γ_*e*_.

### Fabrication

All our meta-devices were fabricated following standard EBL and lift off processes. First, the positive resist was successively spin coated on a silica substrate, and exposed with EBL (JEOL 6300) with an acceleration voltage of 100 kV. After exposure, the samples were developed in the solution of isopropanol alcohol and methyl isobutyl ketone. Then, 3 nm Cr and 30 nm Au/Ag were deposited using electron-beam evaporation. Finally, the top patterns were formed after a lift of process. All samples had dimensions of 80 µm × 80 µm.

### Optical characterizations

We used a homemade macroscopic spectrometer equipped with a broadband supercontinuum white light source and a fiber-coupled grating spectrometer (Ideaoptics NIR2500) to characterize the optical properties of fabricated samples (see more details in Sec. VII of Supplementary Information).

## Supplementary information

Supplementary Material
