# Supplementary material for: Tailoring the lineshapes of coupled plasmonic systems based on a theory derived from first principles
Source: Light Sci Appl. 2020 Sep 8;9:158. doi: 10.1038/s41377-020-00386-5 (PMC7479621; doi:10.1038/s41377-020-00386-5)
Supplement: Supplementary file 1 — Supplementary Material [file 41377_2020_386_MOESM1_ESM.docx]

**Supplementary Information:**

**Tailoring the lineshapes of coupled plasmonic systems based on a theory derived from first principles**

Jing Lin,^1$^ Meng Qiu^1$^, Xiyue Zhang^1$^ , Huijie Guo^1^, Qingnan Cai^1^, Shiyi Xiao^2*^, Qiong He^1,3*^, and Lei Zhou^1,3*^

^1^ State Key Laboratory of Surface Physics, Key Laboratory of Micro and Nano Photonic Structures (Ministry of Education) and Physics Department, Fudan University, Shanghai 200433, China

^2^ Key laboratory of Specialty Fiber Optics and Optical Access Networks, Joint International Research Laboratory of Specialty Fiber Optics and Advanced Communication, Shanghai Institute for Advanced Communication and Data Science, Shanghai University, Shanghai 200444, China

^3^ Collaborative Innovation Center of Advanced Microstructures, Nanjing, 210093, China

**^$^** These authors contributed equally to this work

E-mails: phzhou@fudan.edu.cn, phxiao@shu.edu.cn, qionghe@fudan.edu.cn

1. **Comparisons between LEM and QNM**


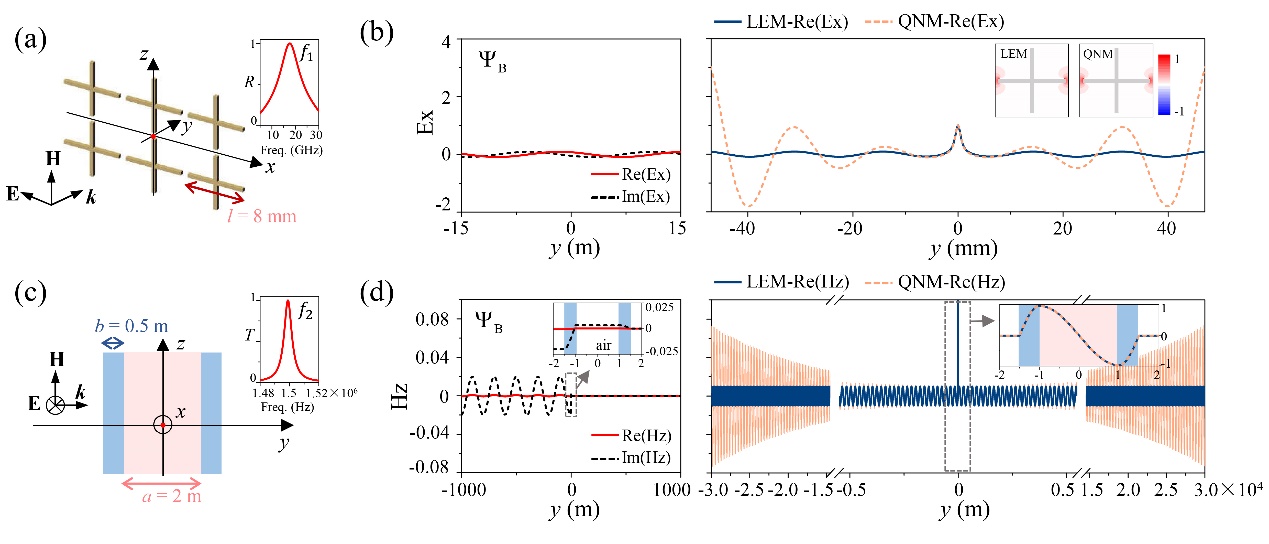


Fig. S1 **Comparisons between LEM and QNM**. **a.** Example 1: a periodic metasurface consisting of array of metallic crosses. Lattice constant is 9 mm and the width and thickness of each metallic wire are 0.5 mm. Inset depicts the calculated reflectance spectrum of the sample. **b.**$E_{x}$distributions of the $\Psi_{\text{B}}$(left panel), LEM (blue) and the QNM (orange) (right panel) for the system described in **a.** Insets depict the field distributions on the yz-plane within one unit cell for the LEM and QEM. **c.** Example 2: an FP cavity formed by two highly reflective dielectric slabs with index $n=100$filled with dielectric material with index $n=50$. Inset depict the computed transmittance spectrum of the system. **d)**$H_{z}$ distributions of the$\Psi_{\text{B}}$ (left panel), the LEM (blue) and the QNM (orange) (right panel) for the system described in **c**). Inset compares the field distributions of two modes in the region near the system. Fields are scaled by their maximum in near-field area in all cases.

To see the clear differences between the leaky eigen mode (LEM) defined in our paper and the quasi normal mode (QNM) defined in Ref. [1], we consider 2 examples --- a metasurface consisting of a periodic array of metallic crosses (Fig. S1a) and an FP cavity (Fig. S1c). We note that these two examples are typical enough since they correspond to transparent background (example 1) and opaque background (example 2), respectively.

We first follow the strategy described in the main text to determine the LEM wavefunctions of the 2 systems. Shining the two systems with normally incident plane waves, we get their response spectra (insets to Figs. S1(a,c)), from which we can easily identify the resonant frequencies ($f_{1}=17.62\text{GHz}$and$f_{2}=1.499\times10^{6}\text{Hz}$) and the total field distributions at these frequencies. We next compute the field distributions $\Psi_{\text{B}}$ in the cases that the “background” systems are shined by the same incident fields. According to the strategy of determining the “background” described in the main text, we find that the background is just air in case 1 (transparent background), but should be the system with inner medium replaced by air in case 2 (opaque background). The “background” of case 2 is chosen in such a way that it not only exhibits the desired (totally reflecting) optical properties but also has the same geometry with the original system. Subtracting the background field distributions (left panels in Figs. S1(b,d)) from the total ones at their resonance frequencies, we then obtain the LEM wave-functions and depict them in Fig. S1(b,d) as blue solid lines. Note that these LEM wave-functions are un-normalized.

We next compute the QNM wavefunctions of the two systems, which are the solutions of source-free Maxwell’s equations $\nabla\times\tilde{E}=i2\pi\tilde{f}_{i}\mu\tilde{H}$and $\nabla\times\tilde{H}=-i2\pi\tilde{f}_{i}\varepsilon\tilde{E}$ with outgoing-radiation boundary conditions. Due to the nature of open system [^1^](#_ENREF_1), [^2^](#_ENREF_2), the eigen-frequencies must be complex values, which in our cases are $\tilde{f}_{1}\text{=(}17.356-i3.6404\text{)GHz}$ and $\tilde{f}_{2}\text{=(}1.499\times10^{6}-i3.181\times10^{3}\text{)Hz}$, respectively. Computed QNM field distributions are shown in Figs. S1(b,d) as orange dashed lines, compared with their LEM counterparts.

Comparisons between two wave-functions reveal their clear differences and connections: 1) Mode frequencies are real for LEM but are complex for QNM, although their real parts are nearly the same; 2) At the infinity ($\left|y\right|\to\infty$), QNM wave-functions diverge exponentially but LEM ones keep constant radiation power inside each channel (we expect the LEM field decay as $r^{-1}$ in 3-dimensional systems); 3) Their near-field distributions are quite similar due to the high-Q nature of the modes. All these features indicate that LEM and QNM are different representations of the modes, obtained under different conditions (e.g., with or without external illuminations), which are suitable for different scenarios. Since in this paper we aim to establish a theory to study the scattering spectrum of the nanophotonic systems, LEM is a natural choice as it is obtained under external illuminations.

1. **Derivation of Eqs. (7-9) in the main text**

Put Eq. (6) to Eq. (1), we get:

$\omega\left[\sum_{q} s_{q}^{+}\Psi_{\text{B}}^{q}\text{+}\sum_{n} a_{n}|\psi_{n}^{\text{LEM}}\rangle\right]=\left(\hat{\mathbf{H}}_{\mathbf{h}}+\sum_{n} \hat{\mathbf{V}}_{n}\right)\left[\sum_{q} s_{q}^{+}\Psi_{\text{B}}^{q}\text{+}\sum_{n} a_{n}|\psi_{n}^{\text{LEM}}\rangle\right]$. (S1)

Projecting both sides of Eq. (S1) by$\langle\psi_{m}^{\text{NF}}|$, we obtain the following equation

$\begin{matrix} \omega a_{m}=\langle\psi_{m}^{\text{NF}}|\hat{\mathbf{H}}_{\mathbf{0}}+\hat{\mathbf{V}}_{m}|\psi_{m}^{\text{LEM}}\rangle_{V}a_{m}\text{+}\langle\psi_{m}^{\text{NF}}|\sum_{n\neq m} \hat{\mathbf{V}}_{n}|\psi_{m}^{\text{LEM}}\rangle_{V}a_{m} \\ +\sum_{n\neq m} \sum_{l\neq n} \langle\psi_{m}^{\text{NF}}|\hat{\mathbf{V}}_{l}|\psi_{n}^{\text{LEM}}\rangle_{V}a_{n}\text{+}\sum_{n} \sum_{q} \langle\psi_{m}^{\text{NF}}|\hat{\mathbf{V}}_{n}|\Psi_{\text{B}}^{q}\rangle_{V}s_{q}^{+} \end{matrix}$, (S2)

where we have used$\left\langle\psi_{m}^{\text{NF}}\right.|\psi_{n}^{\text{NF}}\rangle_{V}\approx\delta_{mn}$,$\left\langle\psi_{m}^{\text{NF}}\right.|\psi_{n}^{\text{FF}}\rangle_{V}\approx0$ and$\left\langle\psi_{m}^{\text{NF}}\right.|\Psi_{\text{B}}^{q}\rangle_{V}\approx0$.

Consider the first term at the right-hand side of Eq. (S2). Using the definition $\psi_{m}^{\text{LEM}}(\vec{r},\omega)=\psi_{m}^{\text{NF}}(\vec{r})+\psi_{m}^{\text{FF}}(\vec{r},\omega)$ which is the extended version of Eq. (2) in the main text for arbitrary frequencies$\omega\neq\omega_{m}$, we obtain that

$\begin{matrix} \langle\psi_{m}^{\text{NF}}|\hat{\mathbf{H}}_{h}+\hat{\mathbf{V}}_{m}|\psi_{m}^{\text{LEM}}\rangle_{V} \\ =\langle\psi_{m}^{\text{NF}}|\hat{\mathbf{H}}_{h}+\hat{\mathbf{V}}_{m}|\psi_{m}^{\text{FF}}\rangle_{V}+\langle\psi_{m}^{\text{NF}}|\hat{\mathbf{H}}_{h}+\hat{\mathbf{V}}_{m}|\psi_{m}^{\text{NF}}\rangle_{V} \\ =\langle\psi_{m}^{\text{NF}}|\hat{\mathbf{H}}_{h}|\psi_{m}^{\text{FF}}\rangle_{V}+\langle\psi_{m}^{\text{NF}}|\hat{\mathbf{V}}_{m}|\psi_{m}^{\text{FF}}\rangle_{V}+\langle\psi_{m}^{\text{NF}}|\hat{\mathbf{H}}_{h}+\hat{\mathbf{V}}_{m}|\psi_{m}^{\text{NF}}\rangle_{V} \end{matrix}$. (S3)

We now separately discuss the three terms in Eq. (S3). For the first term, noting that$|\psi_{m}^{\text{FF}}\rangle$ can be expanded by the FF eigen-modes $\left\{|k_{q}^{\pm}\rangle\right\}$, which are the eigenmodes of $\hat{\mathbf{H}}_{h}$in free-space with frequency$\omega$, we get

$\langle\psi_{m}^{\text{NF}}|\hat{\mathbf{H}}_{h}|\psi_{m}^{\text{FF}}\rangle_{V}=\omega\langle\psi_{m}^{\text{NF}}|\psi_{m}^{\text{FF}}\rangle_{V}$. (S4)

This term can be dropped under the high-Q approximation since $\langle\psi_{m}^{\text{NF}}|\psi_{m}^{\text{FF}}\rangle_{V}<<\langle\psi_{m}^{\text{NF}}|\psi_{m}^{\text{NF}}\rangle_{V}=1$. The second term in Eq. (S3) is non-neglible and can be re-written as

$\langle\psi_{m}^{\text{NF}}|\hat{\mathbf{V}}_{m}|\psi_{m}^{\text{FF}}\rangle_{V}\approx-i\Gamma_{m}$. (S5)

with $\Gamma_{m}$ being a real number describing the radiation damping of this mode. That $\langle\psi_{m}^{\text{NF}}|\hat{\mathbf{V}}_{m}|\psi_{m}^{\text{FF}}\rangle_{V}$ is approximately a purely imaginary number can be understood by considering the rigorous solution of a radiating dipole, which shows that there must be a$\pi/2$ phase difference between $\psi_{m}^{\text{FF}}$ and $\psi_{m}^{\text{NF}}$ at the vicinity of the dipole [^3^](#_ENREF_3).

Finally, considering that $\psi_{m}^{\text{NF}}$ is the approximate eigenmode of the single-resonator Hamiltonian (see Eq. (3) in the main text), for the third term in Eq. (S3) we obtain that

$\langle\psi_{m}^{\text{NF}}|\hat{\mathbf{H}}_{h}+\hat{\mathbf{V}}_{m}|\psi_{m}^{\text{NF}}\rangle_{V}\approx\omega_{m}\langle\psi_{m}^{\text{NF}}|\psi_{m}^{\text{NF}}\rangle_{V}\approx\omega_{m}$. (S6)

Now we consider the remaining terms in Eq. (S2). Since the NF wave-functions are well bounded at the vicinities of different resonators under the high-Q condition, we find that $\hat{\mathbf{V}}_{n}|\psi_{m}^{\text{LEM}}\rangle_{V}$ are negligible as long as$n\neq m$, and thus we simplify those terms as

$\left\{\begin{matrix} \langle\psi_{m}^{\text{NF}}|\sum_{n\neq m} \hat{\mathbf{V}}_{n}|\psi_{m}^{\text{LEM}}\rangle_{V}\approx0 \\ \sum_{l\neq n} \langle\psi_{m}^{\text{NF}}|\hat{\mathbf{V}}_{l}|\psi_{n}^{\text{LEM}}\rangle_{V}\approx\langle\psi_{m}^{\text{NF}}|\hat{\mathbf{V}}_{m}|\psi_{n}^{\text{LEM}}\rangle_{V}=\langle\psi_{m}^{\text{NF}}|\hat{\mathbf{V}}_{m}|\psi_{n}^{\text{NF}}\rangle_{V}+\langle\psi_{m}^{\text{NF}}|\hat{\mathbf{V}}_{m}|\psi_{n}^{\text{FF}}\rangle_{V} \\ \sum_{n} \langle\psi_{m}^{\text{NF}}|\hat{\mathbf{V}}_{n}|\Psi_{\text{B}}^{q}\rangle_{V}\approx\langle\psi_{m}^{\text{NF}}|\hat{\mathbf{V}}_{m}|\Psi_{\text{B}}^{q}\rangle_{V} \end{matrix}\right.$. (S7)

Collecting all terms, we finally derive Eq. (S2) as

$\begin{matrix} \omega a_{m}=(\omega_{m}-i\Gamma_{m})a_{m}+\sum_{n\neq m} \left(\langle\psi_{m}^{NF}|\hat{\mathbf{V}}_{m}|\psi_{n}^{NF}\rangle_{V}+\langle\psi_{m}^{NF}|\hat{\mathbf{V}}_{m}|\psi_{n}^{FF}\rangle_{V}\right)a_{n} \\ \text{ +}\sum_{q} \langle\psi_{m}^{NF}|\hat{\mathbf{V}}_{m}|\Psi_{\text{B}}^{q}\rangle_{V}s_{q}^{+} \end{matrix}$, (S8)

which can be re-written as the following parametrized form:

$\omega a_{m}=(\omega_{m}-i\Gamma_{m})a_{m}+\sum_{n\neq m} (t_{mn}+iX_{mn})a_{n}\text{+}\sum_{q} i\kappa_{mq}s_{q}^{+}$ (S9)

with

$\left\{\begin{matrix} \Gamma_{m}=i\langle\psi_{m}^{\text{NF}}|\hat{\mathbf{V}}_{m}|\psi_{m}^{\text{FF}}\rangle_{V} \\ t_{mn}=\langle\psi_{m}^{\text{NF}}|\hat{\mathbf{V}}_{m}|\psi_{n}^{\text{NF}}\rangle_{V} \\ X_{mn}=-i\langle\psi_{m}^{\text{NF}}|\hat{\mathbf{V}}_{m}|\psi_{n}^{\text{FF}}\rangle_{V} \\ \kappa_{mq}=-i\langle\psi_{m}^{\text{NF}}|\hat{\mathbf{V}}_{m}|\Psi_{\text{B}}^{q}\rangle_{V} \end{matrix}\right.$. (S10)

Here, again we used the fact that $\psi_{m}^{\text{FF}}$ and $\psi_{m}^{\text{NF}}$ exhibit a $\pi/2$ phase difference at the origin where the resonator is placed. Multiply $-i$ on both sides of Eq. (S9), we finally get Eq. (7) in the main text.

Similarly, project both sides of Eq. (6) in the main text by $\langle k_{q}^{-}|$, we obtain:

$\begin{matrix} s_{q}^{-}\text{=}\langle k_{q}^{-}|\Psi(\vec{r},\omega)\rangle_{S}=\sum_{p} s_{p}^{+}\langle k_{p}^{-}|\Psi_{\text{B}}^{p}\rangle_{S}\text{+}\sum_{n} a_{n}\langle k_{q}^{-}|\psi_{n}^{\text{LEM}}\rangle_{S} \\ =\sum_{p} s_{p}^{+}\langle k_{p}^{-}|\Psi_{\text{B}}^{p}\rangle_{S}\text{+}\sum_{n} a_{n}\langle k_{q}^{-}|\psi_{n}^{\text{FF}}\rangle_{S}\text{+}\sum_{n} a_{n}\langle k_{q}^{-}|\psi_{n}^{\text{NF}}\rangle_{S} \end{matrix}$, (S11)

where the integrals are performed on each reference *plane* of different external port. Under the high-*Q* approximation, we can set $\langle k_{q}^{-}|\psi_{n}^{\text{NF}}\rangle_{S}\approx0$ since the NF wave-functions are well bounded at the vicinities of resonators and decay to 0 at the port. Thus we get

$s_{q}^{-}=\sum_{p} s_{p}^{+}\langle k_{q}^{-}|\Psi_{\text{B}}^{p}\rangle_{S}\text{+}\sum_{n} a_{n}\langle k_{q}^{-}|\psi_{n}^{\text{FF}}\rangle_{S}$ (S12)

which can be re-written as Eq. (8) in the main text with

$\left\{\begin{matrix} c_{qp}=\langle k_{q}^{-}|\Psi_{\text{B}}^{p}\rangle_{S} \\ d_{qm}=\langle k_{q}^{\text{-}}|\psi_{m}^{\text{FF}}\rangle_{S} \end{matrix}\right.$. (S13)

Thus, we have successfully derived Eq. (7-9) in the main text.

1. **Formalisms for photonic systems**

According to the Hamiltonian form of photonic systems (Eq. (10) in the main text), we find that the potential operator contributed by the *m*-th scatter is[^4^](#_ENREF_4), [^5^](#_ENREF_5)

$\hat{\mathbf{V}}_{m}=\hat{\mathbf{H}}_{m}-\hat{\mathbf{H}}_{h}\text{=}\left(\begin{matrix} 0 & -i\left(\frac{1}{\mu_{m}}-\frac{1}{\mu_{h}}\right)\nabla\times& 0 & 0 \\ i\left(\frac{1}{\varepsilon_{\infty,m}}-\frac{1}{\varepsilon_{\infty,h}}\right)\nabla\times& 0 & 0 & -i\left(\frac{1}{\varepsilon_{\infty,m}}-\frac{1}{\varepsilon_{\infty,h}}\right) \\ 0 & 0 & 0 & i \\ 0 & i\left(\omega_{p,m}^{2}\varepsilon_{\infty,m}-\omega_{p,h}^{2}\varepsilon_{\infty,h}\right) & -i\left(\omega_{0,m}^{2}-\omega_{0,h}^{2}\right) & 0 \end{matrix}\right)$ (S14)

where$\varepsilon_{\infty,m}$, $\mu_{m}$ and $\omega_{0,m}$ are position-dependent functions describing the properties of the *m*-th scatter, and $\varepsilon_{\infty,h}$, $\mu_{h}$, and $\omega_{0,h}$ describe the homogeneous host medium. Here, we first consider the lossless case (i.e.,$\Gamma_{e}=0$). Further consider a special case that scatters formed by plasmonic metals (Au or Ag) and the host medium is just air, then we have$\varepsilon_{\infty,m}\text{=}\varepsilon_{\infty,h}=\varepsilon_{0}$, and thus Eq. (S14) can be simplified as

$\hat{\mathbf{V}}_{m}=\left(\begin{matrix} 0 & 0 & 0 & 0 \\ 0 & 0 & 0 & 0 \\ 0 & 0 & 0 & i \\ 0 & i\omega_{p,\mathrm{m}}^{2}\varepsilon_{0} & 0 & 0 \end{matrix}\right)$, (S15)

After obtaining the wave-functions $\{\psi_{m}^{\text{NF}},\psi_{m}^{\text{FF}}\}$ for all individual resonators and using the inner product defined in Eqs. (11,12) in the main text for photonic problems, we get the following explicit forms of those parameters defined in Eq. (9) for plasmonic systems as

$\left\{\begin{matrix} \Gamma_{m}=-i\omega_{m}\int_{V_{m}} dr\left[\vec{P}_{m}^{*}\cdot\vec{E}_{m}^{FF}\right]/2 \\ \kappa_{mq}=-\omega_{m}\int_{V_{m}} dr\left[\vec{P}_{m}^{*}\cdot\vec{E}_{q}^{+}\right]/2 \\ d_{qm}=\frac{1}{2}\oint[\mu_{0}\vec{H}_{q}^{-*}\cdot\vec{H}_{m}^{FF}+\varepsilon_{h}\vec{E}_{q}^{-*}\cdot\vec{E}_{m}^{FF}]\vec{c}\cdot d\vec{S} \end{matrix}\right.,\text{ }\left\{\begin{matrix} t_{mn}\text{=}-\omega_{m}\int_{V_{m}} dr\left[\vec{P}_{m}^{*}\cdot\vec{E}_{n}^{NF}\right]/2 \\ X_{mn}=i\omega_{m}\int_{V_{m}} dr\left[\vec{P}_{m}^{*}\cdot\vec{E}_{n}^{FF}\right]/2 \\ c_{qp}=\frac{1}{2}\oint[\mu_{0}\vec{H}_{q}^{-*}\cdot\vec{H}_{p}^{+}+\varepsilon_{h}\vec{E}_{q}^{-*}\cdot\vec{E}_{p}^{+}]\vec{c}\cdot d\vec{S} \end{matrix}\right.$, (S16)

where we have defined $\vec{P}_{m}(\vec{r})\approx\left[\varepsilon_{m}(\omega_{m})-\varepsilon_{0}\right]\vec{E}_{m}^{\text{NF}}(\vec{r})$ to describe the distribution of polarization density inside the *m*-th plasmonic resonator.

Now we consider the lossy cases. The perturbation caused by absorption can be defined as $\hat{\mathbf{H}}_{m}^{\text{a}}-\hat{\mathbf{H}}_{m}^{0}=\text{diag}\left(\begin{matrix} 0 & 0 & 0 & -i\Gamma_{e} \end{matrix}\right)$[^4^](#_ENREF_4),[^5^](#_ENREF_5). Then we can get the absorption damping as

$\Gamma_{m}^{a}=i\langle\psi_{m}^{\text{NF}}|\hat{\mathbf{H}}_{m}^{a}-\hat{\mathbf{H}}_{m}^{0}|\psi_{m}^{\text{NF}}\rangle_{V}=\int_{V_{m}} dr\left(\frac{\omega_{p}^{2}}{\omega_{m}^{3}}\Gamma_{e}\varepsilon_{0}\right)\frac{1}{2}\omega_{m}\left|\vec{E}_{m}^{\text{NF}}\right|^{2}\approx\mathrm{Im}[\varepsilon\left(\omega_{m}\right)]\frac{1}{2}\omega_{m}\int_{V_{m}} dr\left|\vec{E}_{m}^{\text{NF}}\right|^{2}$ , (S17)

with$\mathrm{Im}(\varepsilon)\text{=}\frac{\omega_{p}^{2}}{\omega_{m}\left(\omega_{m}^{2}\text{+}\Gamma_{e}^{2}\right)}\Gamma_{e}\varepsilon_{0}\approx\frac{\omega_{p}^{2}}{\omega_{m}^{3}}\Gamma_{e}\varepsilon_{0}$.

Finally, we note that in general cases where the host medium is not air and/or the resonators are not formed by good plasmonic metals, one needs to re-derive the formulas to calculate these parameters.

1. **Analytical Mie-solutions of a single Au sphere**

Consider a gold sphere with radius $r_{m}$ and permittivity $\varepsilon(\omega)=\varepsilon_{0}[1-\omega_{p}^{2}/\omega^{2}]$ placed at the origin, shined by external plane waves. Such a scattering problem can be analytically solved by the Mie theory[^6^](#_ENREF_6), yielding an analytical form of the total response wave-function$\Psi^{\text{res}}(\vec{r},\omega)$. Specifically, the total electric-field of such a scattering problem can be generally expanded as

$\vec{E}^{\text{res}}=\sum_{l=1}^{\infty} [a_{l,TE}\nabla\times(\vec{r}A_{l,TE})-a_{l,TM}(i/k)\nabla\times\nabla\times(\vec{r}A_{l,TM})]$, (S18)

with

(S19)

where $k=k_{0}\text{=}\omega\sqrt{\mu_{0}\varepsilon_{0}}$ and $k=k_{m}\text{=}\omega\sqrt{\mu_{0}\varepsilon(\omega)}$ are the wave vectors of light inside and outside the sphere, respectively, $a_{l,\sigma}$are expansion coefficients determined by matching boundary conditions, $\sigma\text{=TE(or TM)}$denotes the polarization that the electric (or magnetic) field is perpendicular to $\vec{r}$, , and $P_{l}^{1}$ is the associated Legendre polynomial. The$R_{l}(kr)$function takes the form of Hankel function of the first kind $h_{l}^{(1)}(k_{0}r)$outside the sphere, and spherical Bessel function $j_{l}(k_{m}r)$ inside the sphere.

In the frequency region where $r_{m}$ is much smaller than the resonant wavelength, the electric dipole channel $\text{TM}_{1}$($l=1,\sigma=\text{TM}$) dominates the response[^7^](#_ENREF_7). Under such approximation, we get

(S20)

where $\hat{J}_{l}(x)=x\cdot j_{l}(x)$ and$\hat{H}_{l}^{(1)}(x)=x\cdot h_{l}^{(1)}(x)$. We can find the frequency at which the response diverges through finding the pole of the scattering coefficient:

. (S21)

Solution of Eq. (S21) is in general a complex value, and the resonant frequency is the real part of it. Expanding all involved terms in Eq. (S21) to Taylor series in$r_{m}/\lambda_{p}$, we finally obtain an analytical expression of the resonant frequency

$\omega_{\text{res}}=[1-8\pi^{2}(r_{m}/\lambda_{p})^{2}/15+...]\omega_{p}/\sqrt{3}$. (S22)

Following the general strategy defined in the main text, set $\omega$ as $\omega_{res}$ and then extract the background wave-function (i.e., the incident field) from the total field distribution, we finally get the analytical form the LEM wave-function as

(S23)

where $E_{m}$ is a normalization constant. In principle, the field distribution inside the sphere in above equation also includes the incident field and should be subtracted. In order to make the expression concise, we ignore this operation which is justified by the high-Q approximation.

We follow the general strategy described in the main text to determine far-field (FF) wave-function$\vec{E}_{m}^{\text{FF}}$. In the limit of$r\to\infty$, we find that$h_{1}^{(1)}(k_{0}r)\to-e^{ik_{0}r}$. Put this form into Eq. (S23), we identify $\vec{E}_{m}^{\text{FF}}$as

(S24)

and get the near-field wave-function subsequently by

$\vec{E}_{m}^{\text{NF}}(\vec{r})=\vec{E}_{m}^{\text{LEM}}(\vec{r})-\vec{E}_{m}^{\text{FF}}(\vec{r})$. (S25)

Equations (S23-25) are the E-field parts of $\{\psi_{m}^{\text{LEM}}(\vec{r}),\psi_{m}^{\text{FF}}(\vec{r}),\psi_{m}^{\text{NF}}(\vec{r})\}$ for the nano sphere. Other field components $\{\vec{H},\vec{P},\vec{V}\}$ can be determined in a similar way. We emphasize that although the field at the origin is divergent under this definition, the integrations defined in Eq. (9) all exhibit finite values.

We next illustrate how to expand $\Psi_{\text{B}}$in terms of the port modes. As we mentioned before, the channel $\text{TM}_{1}$ dominates the scatterings and thus the problem under study is essentially a single-port system. To determine the port mode for this channel, we note that the exact solution of the $\text{TM}_{1}$ channel is . Using the approximation that $h_{l}^{(1\text{，}2)}(k_{0}r)\to(\mp\mathrm{i})^{l+1}e^{\pm ik_{0}r}$ as$r\to\infty$, we finally get the port mode as

(S26)

where $l=1$and$E_{q}\text{=}\sqrt{3\pi E_{0}^{2}/(2\eta_{0}k_{0}^{2})}$ is a constant to normalize the incident energy flow with$E_{0}$ being the incident field. The magnetic field $\vec{H}_{q}^{\pm}$ can be determined in a similar way and we get the complete form of the FF eigen wavefunctions $|k_{q}^{\pm}\rangle=\left(\begin{matrix} \vec{H}_{q}^{\pm} & \vec{E}_{q}^{\pm} & 0 & 0 \end{matrix}\right)^{T}$. Project $|k_{q}^{\pm}\rangle$ onto the background wave$\Psi_{\text{B}}$ (i.e. the incident *x*-polarized plane wave), one can identify $s_{q}^{\pm}=\langle k_{q}^{\pm}|\Psi_{\text{B}}\rangle$ as the incoming (or outgoing) wave amplitudes corresponds different external ports.

Under the excitations of *x*-polarized plane waves with$E_{0}\text{=}1$, we find that $\vec{P}(\vec{r})$ field inside the sphere is almost a constant (see inset to Fig. 2a in the main text), which can help us further simplify the above expressions. Approximate the sphere as a dipole with moment $\vec{p}\approx\int_{\text{sphere}} \vec{P}_{m}(\vec{r})d\vec{r}$ placed at the origin, we find that $\psi_{m}^{\text{FF}}(\vec{r})$ is exactly the far-field radiation of this radiating dipole, which can be re-written as

$\psi_{m}^{\text{FF}}(r)=\left(\begin{matrix} \frac{\omega_{res}^{2}}{4\pi c_{0}r}\hat{e}_{r}\times[\vec{p}], & \frac{\omega_{res}^{2}}{4\pi\varepsilon_{0}c_{0}^{2}r}\hat{e}_{r}\times\left(\hat{e}_{r}\times[\vec{p}]\right), & 0, & 0 \end{matrix}\right)^{T}$. (S27)

Put this formula to Eq. (S16), we find that

$\begin{matrix} \Gamma=-i\omega_{\text{res}}\int_{V} dr\left[\vec{P}^{*}\cdot\vec{E}^{FF}\right]/2 \\ \approx\frac{1}{2}\omega_{\text{res}}\vec{p}\cdot\lim_{r_{m}\to0} \left(\mathrm{Im}\{\int_{V} dr\vec{E}_{x}^{FF}\}/(\frac{4}{3}\pi r_{m})\right)_{r_{m}\to0}=\frac{p^{2}\omega_{res}^{4}}{12\pi\varepsilon_{0}c_{0}^{3}} \end{matrix}$. (S28)

Similarly, put Eq. (S18) to Eq. (S16), we get

$\begin{matrix} \kappa\approx\frac{1}{2}i\omega_{\text{res}}\vec{p}\cdot\lim_{r_{m}\to0} \left(\int_{V} dr\vec{E}_{\text{B}}/(\frac{4}{3}\pi r_{m})\right)_{r_{m}\to0}=\frac{p\omega_{res}^{2}}{c_{0}\sqrt{6\pi c_{0}\varepsilon_{0}}}i \\ d=\lim_{\vec{r}\to\infty} \frac{1}{2}\oint[\mu_{0}\vec{H}_{q}^{-*}\cdot\vec{H}^{FF}+\varepsilon_{h}\vec{E}_{q}^{-*}\cdot\vec{E}^{FF}]\vec{c}\cdot d\vec{S}=\frac{p\omega_{res}^{2}}{c_{0}\sqrt{6\pi\varepsilon_{0}c_{0}}}i \end{matrix}$. (S29)

Thus, comparisons between Eqs. (S28) and (S29) show that we have successfully derived out Eq. (13) in the main text.

1.
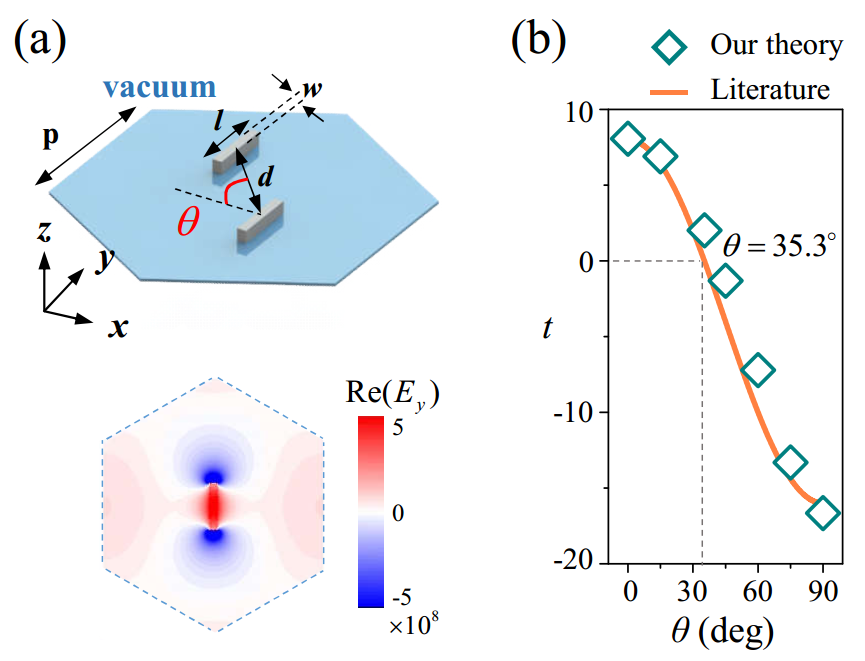
**Justifying our theory against an analytical formula on dipolar interactions**

Fig. S2 **a**. Schematics of the plasmonic system under study with geometrical parameters *p*=530, *w*=30, *l*=200, all in units of nm. We vary the relative angle $\theta$ between two resonators with distance *d* fixed as 270 nm. Lower panel depicts the LEM wavefunction (distribution of $\mathrm{Re}(E_{y})$on the$xoy$plane) of a single bar. **b**. Inter-resonator coupling strength $t$ as a function of $\theta$ calculated by our theory (open squares) and the analytical formula derived in prior literature (solid line).

Consider a series of metasurfaces consisting of periodic arrays of 2 coupled Ag bars arranged in different configurations. From the computed field distribution of a single bar shown in Fig. S2a, we see clearly that the bar resonator supports an electric-dipole ($\vec{p}$) mode. Since the periodicity of our metasurface is quite large, the system well represents two isolated coupled dipoles$\vec{p}$. We use our theory (i.e., Eq. (9) in the main text) to compute the NF coupling strengths *t* between two such resonators under different configurations (with different $\theta$), and the results are shown in Fig. S2b as open symbols.

Meanwhile, it has been demonstrated previously (Refs. 45 and 46 in the main text) that the NF coupling between two electric dipoles satisfies the following analytical relation:

$t=Ap^{2}(1-3\cos^{2}\theta)$, (S30)

with $A$ being a constant depending on *d* and the NF properties of the dipole mode. In particular, Eq. (S30) predicts that $t=0$ at the magic angle $\theta=35.3^{\circ}$. We see clearly that $t$ computed by our theory match very well with the analytical formula (Fig. S2(b)).

1. **More details on the coupled systems studied in Figs. 3 & 5**


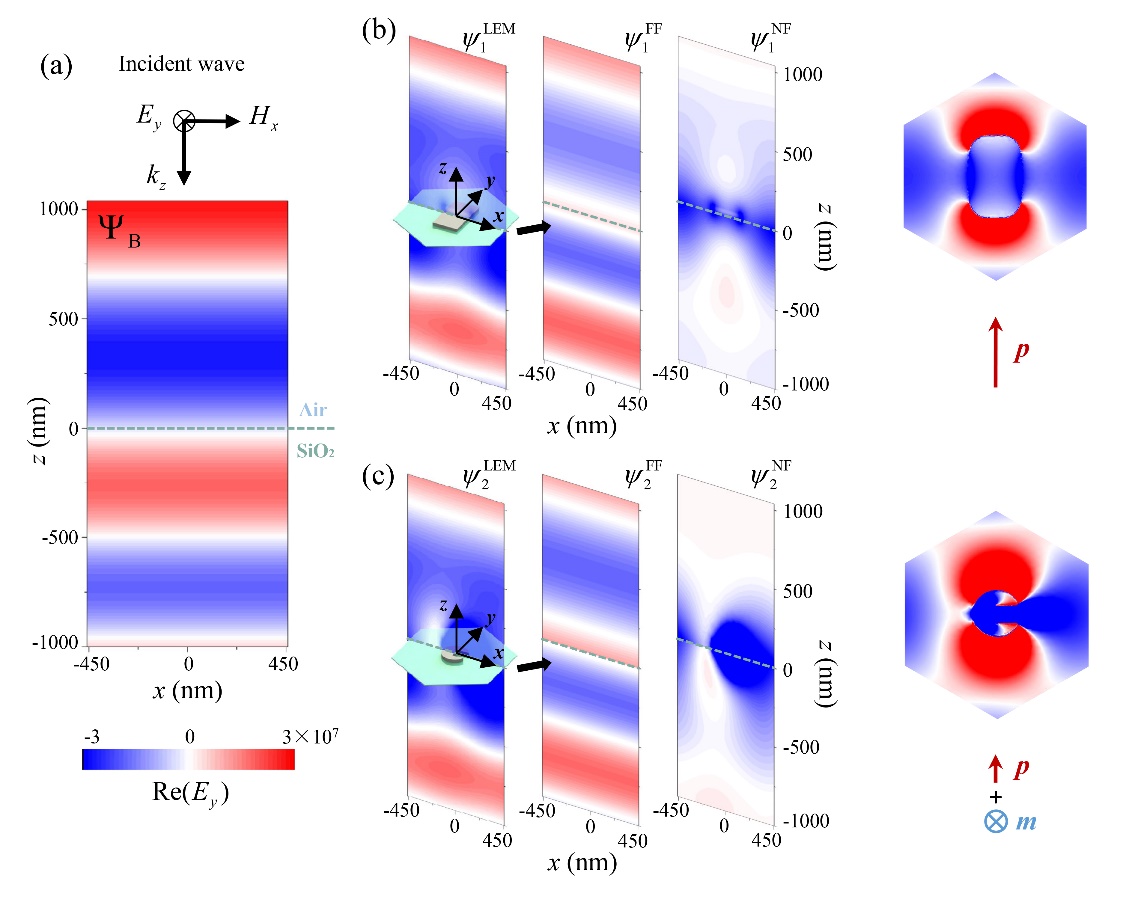


Fig. S3 **Wavefunctions of two basic resonators studied in the main text**. **a**. For the matasurface system studied in the main text, FEM-computed wave-functions $\Psi_{\text{B}}$ at frequency$\omega=2\pi\times207.24\text{THz}$.**b**. For the C-shaped resonator studied in the main text, FEM-computed basic wave-functions {$\psi_{m}^{\text{LEM}}$,$\psi_{m}^{\text{FF}}$and $\psi_{m}^{\text{NF}}$} at its resonance frequency ($\omega_{1}\text{=2}\pi\times207.24\text{THz}$). **c**. For the Bar resonator studied in the main text, FEM-computed basic wave-functions {$\psi_{m}^{\text{LEM}}$,$\psi_{m}^{\text{FF}}$and $\psi_{m}^{\text{NF}}$} at its resonance frequency ($\omega_{2}\text{=2}\pi\times201.71\text{THz}$). Here, we only depict the distributions of $E_{y}$ on the $xoy$ plane. The green dash line represents the air/SiO_2_ interface.

|  | $\Gamma$ | $\Gamma_{i}$ | $\kappa$ | $d$ |
| --- | --- | --- | --- | --- |
| Bar | 17.919-1.081i | 4.168 | -0.422+3.769i | 0.816+3.633i |
| C-shape | 3.176-0.640i | 5.738 | -0.052+1.560i | -0.197+1.620i |

**Table S1. Parameters of two basic resonators, computed by our theory using the wave-functions presented in Fig. S3. Here, units of $\Gamma$and $\Gamma_{i}$are all THz, and units of $\kappa$ and $d$are all (THz)^1/2^ .**

| $\theta$ | $t_{12}$ | $t_{21}$ | $t_{11}$ | $t_{22}$ | $X_{12}$ | $X_{21}$ |
| --- | --- | --- | --- | --- | --- | --- |
| 0 | 19.229+1.549i | 16.213-1.33i | -0.491 | -2.525 | -7.561-1.186i | -7.313+1.051i |
| 23.2 | 13.976+1.123i | 11.098-0.924i | -0.495 | -1.939 | -7.561-1.186i | -7.313+1.051i |
| 50.9 | 3.109+0.243i | 0.506+0.149i | -0.238 | -0.287 | -7.561-1.186i | -7.313+1.051i |
| 60.9 | 0.194+0.007i | -2.126+0.442i | -0.320 | -0.548 | -7.561-1.186i | -7.313+1.051i |
| 70.1 | -3.469-0.290i | -5.630+0.64i | -0.718 | -1.182 | -7.561-1.186i | -7.313+1.051i |
| 90 | -7.133-0.587i | -8.651+0.678i | -1.499 | -1.920 | -7.561-1.186i | -7.313+1.051i |

**Table S2. Coupling parameters between two resonators placed under different relative configurations specified by the angle$\theta$. Units of these parameters are all THz.** For simplify, we take $t=\mathrm{Re}(t_{12})$defined in main text. $t_{ii}\text{=}\langle\psi_{i}^{\text{NF}}|\hat{\mathbf{V}}_{j\neq i}|\psi_{i}^{\text{NF}}\rangle_{V}(i,j=1,2)$is the on-site adjustment to the corresponding eigen frequency$f_{i}$.

Following the general strategy established in the paper we can easily get the single-resonator properties (i.e.,$\{\omega_{m},\psi_{m}^{\text{NF}},\psi_{m}^{\text{FF}}\}$) of two resonators studied in Figs. 3 and 5. Note that the background system these cases is just a single air/SiO_2_ interface, therefore, $\psi_{\text{B}}$ can be easily calculated assuming that a plane wave strikes such an interface (see Fig. S3(a)). Based on the computed single-resonator wave-functions presented in Fig. S3, we can calculate all parameters related to these two modes defined in our theory. These results are shown in Table. S1. Furthermore, with all wave-functions of two resonators completely known (see Fig. S3), we continue to calculate the coupling parameters ($t$and$X$) between two resonators, which are placed at different relative positions specified by the angle $\theta$ (see Fig. 5a in the main text). The results are shown in Table S2. We note that X is a constant since the modes lie on the same phase plane.

We present a simple picture to explain the underlying physics of the BIC discovered in the main text. We diagonalize the matrix containing $t$ in Eq. (14) by an orthogonal transformation$\mathbf{M}$, i.e.

$\mathbf{M}\left(\begin{matrix} \omega_{1} & t \\ t & \omega_{2} \end{matrix}\right)\mathbf{M}^{\text{-}1}\text{=}\left(\begin{matrix} \tilde{\omega}_{\text{+}} & 0 \\ 0 & \tilde{\omega}_{-} \end{matrix}\right)$. (S31)


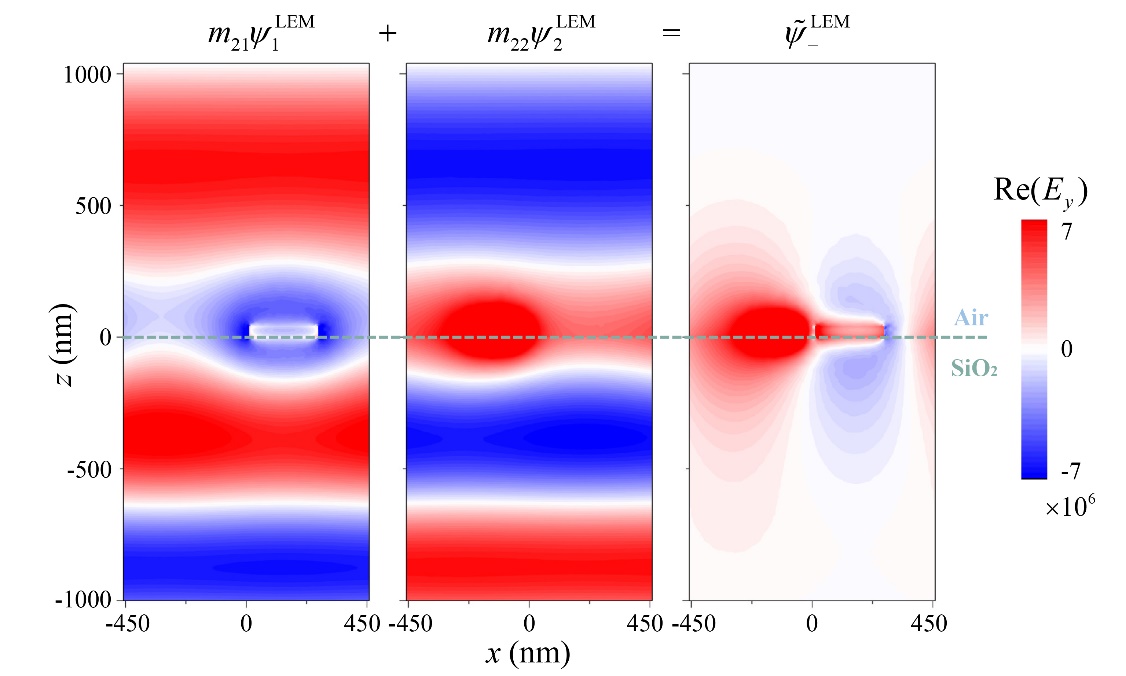


Fig. S4 **Physical mechanism of the BIC.** FEM-computed$E_{y}$-field distributions of the sub-system containing the bar resonators only, the sub-system containing the C-resonators only, and the whole system, at the BIC frequency for sample 3.

We find that

$\mathbf{M}\text{=}\left(\begin{matrix} m_{11} & m_{12} \\ m_{21} & m_{22} \end{matrix}\right)=\left(\begin{matrix} \frac{\Delta\omega+\sqrt{\Delta\omega^{2}+t^{2}}}{\sqrt{t^{2}+\left(\Delta\omega+\sqrt{\Delta\omega^{2}+t^{2}}\right)^{2}}} & \frac{t}{\sqrt{t^{2}+\left(\Delta\omega+\sqrt{\Delta\omega^{2}+t^{2}}\right)^{2}}} \\ \frac{\Delta\omega-\sqrt{\Delta\omega^{2}+t^{2}}}{\sqrt{t^{2}+\left(\Delta\omega-\sqrt{\Delta\omega^{2}+t^{2}}\right)^{2}}} & \frac{t}{\sqrt{t^{2}+\left(\Delta\omega-\sqrt{\Delta\omega^{2}+t^{2}}\right)^{2}}} \end{matrix}\right)$. (S32)

with$\Delta\omega=(\omega_{1}-\omega_{2})/2$. The wave-function of the “dressed” mode is a linear combination of two original modes, $\tilde{\psi}_{-}^{\text{LEM}}=m_{21}\psi_{1}^{\text{LEM}}+m_{22}\psi_{2}^{\text{LEM}}$, with $m_{21}$ and $m_{22}$ being two coefficients (see Eq. (S31)). Tuning the value of *t* can dramatically change the values of $m_{21}$ and $m_{22}$ and thus modify the total radiation of the “dressed” mode. At the BIC frequency, we find $t=3.109$and thus $m_{21}=-0.409$ and $m_{22}\text{=0.912}$, which finally make the radiations from two original modes completely cancel each other (ses Fig. S4) leading to the BIC.

1. **Experimental setup of home-made macroscopic spectrometer**


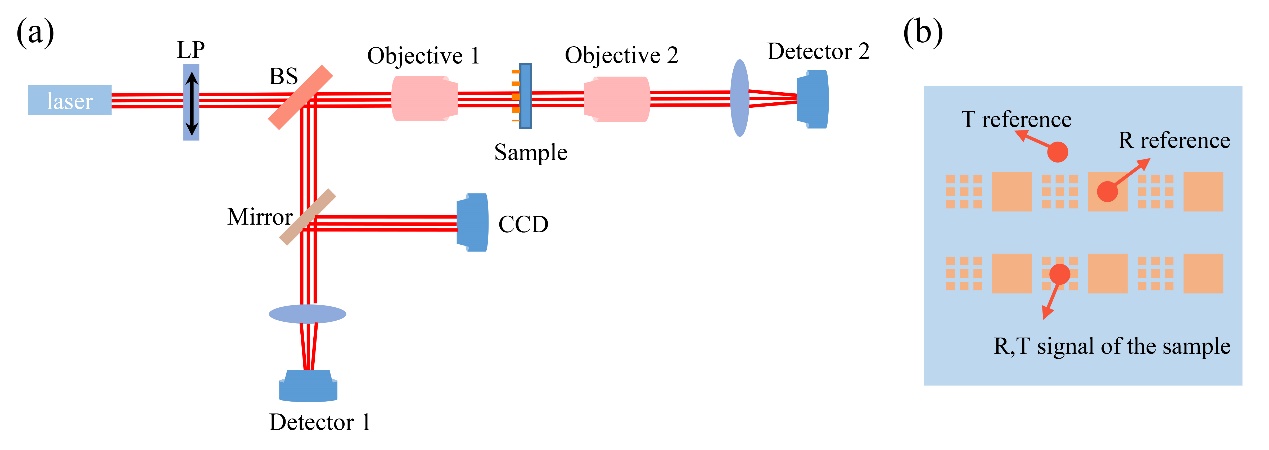


Fig. S5 **Sketch of out experimental setup. a.** Schematic of our home-made macroscopic spectrometer. LP: Linear Polarizer, BS: Beam Splitter. **b.** Schematic of the sample used in experiment containing both the fabricated metasurfaces and metallic patches, based on which we can measure both transmission and reflection spectra of the metasurfaces.

1. **Transmission spectra of all samples obtained by measurements, simulations and our theory**


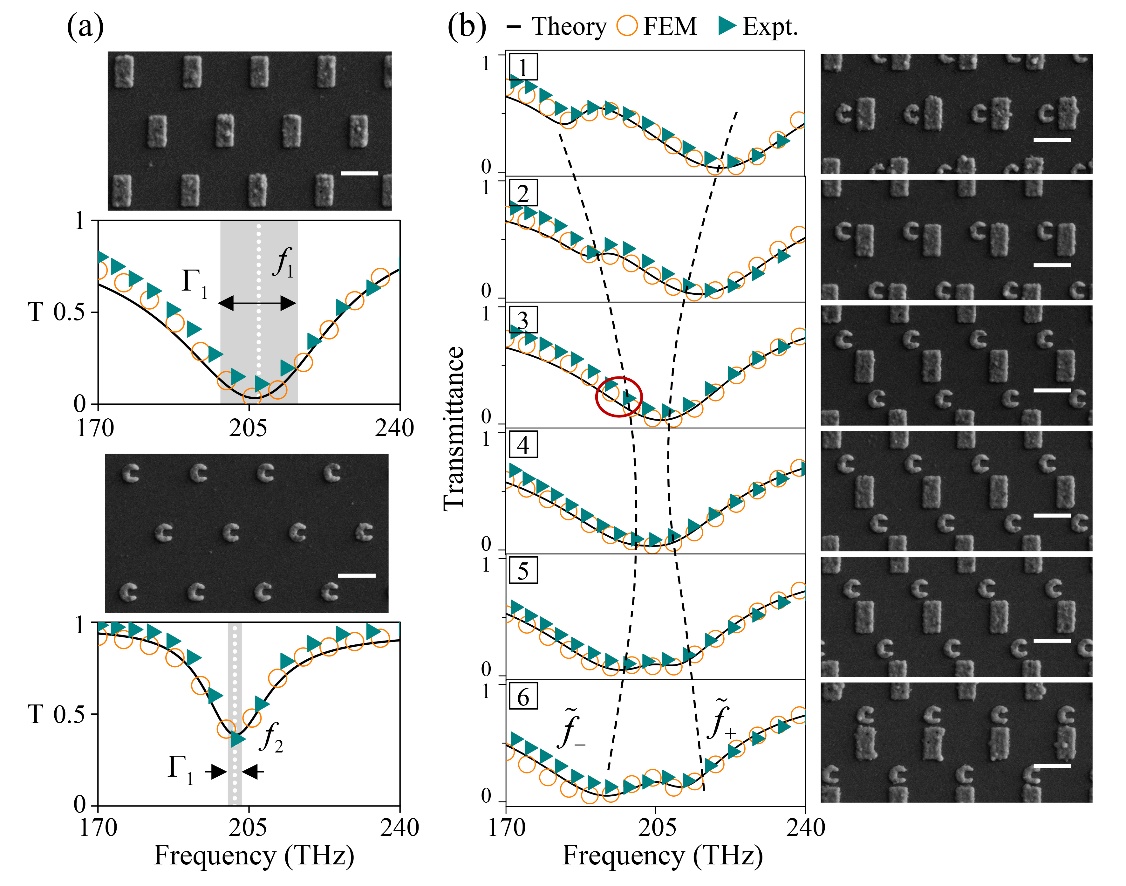


Fig. S6 Transmittance spectra of **a.** periodic metasurfaces containing the bar-resonators and the C-resonators respectively and **b.** 6 systems obtained by our theory (lines), FEM simulations (open circles), and experimental measurements (solid triangles) on realistic samples with SEM pictures shown with the scale bars (white lines), 500 nm.

1.
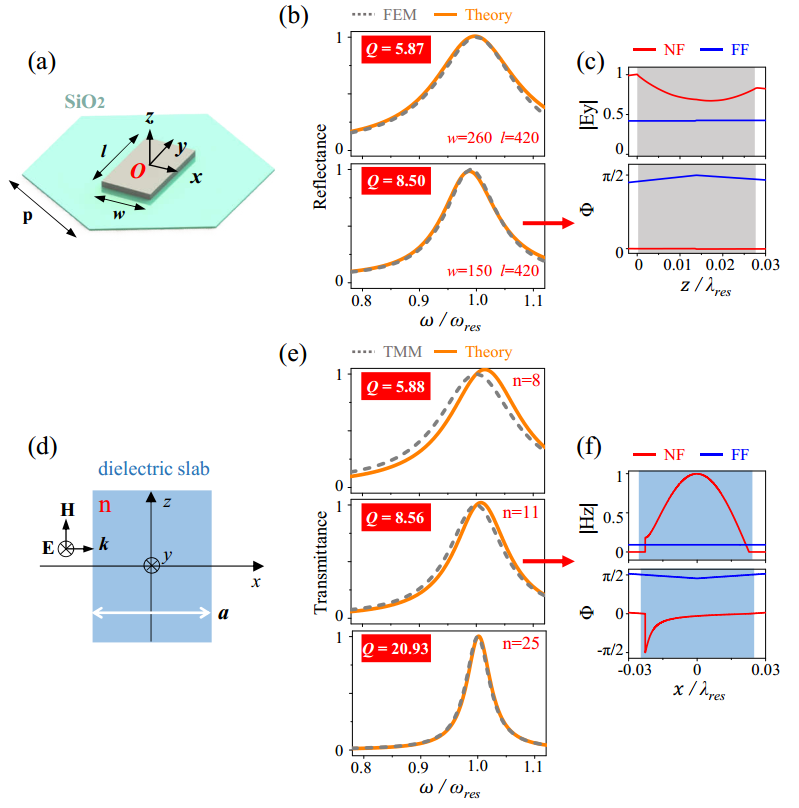
**Validities of our theory in studying resonances of different types**

Fig. S7 **Comparisons between plasmonic systems and dielectric systems**. **a.** Example 1: a periodic metasurface consisting of metallic bars. **b.** Reflectance spectra obtained by our theory (solid lines) and FEM simulations (dashed lines). Geometrical parameters are *w*=260, *l*=420 (upper panel), *w*=150, *l*=420 (lower panel), thickness of metals are both *h*=40 and periodicities are *p*=530, all in units of nm. **c.** Distributions of amplitude (upper) and phase (lower) of $E_{y}$of the FF (blue) and NF (red) wave-functions for the system described in **a.** Gray area represents the interior of the scatterer. **d.** Example 2: a dielectric slab with thickness$a=2\text{m}$. **e.** Reflectance spectra of the systems with different index *n* obtained by our theory (solid lines) and the transfer-matrix-method (TMM) (dashed lines). **f.** $H_{z}$distributions of the FF (blue) and NF (red) wave-functions for the system described in **d**. Blue area represents the interior of the slab. $\omega_{res}$and $\lambda_{res}$represent the resonance frequency and resonance wavelength of the systems under study.

To discuss the validities of our theory, we purposely studied two series of systems containing resonators of different types --- metallic bars exhibiting different shapes (Fig. S7a-c) and dielectric slabs with different refraction index (*n*) (Fig. S7d-f). We note that these two series of examples are typical enough since they represent resonances not only of different nature and also exhibiting different backgrounds.

For plasmonic resonators, our theory work very well (Fig. S7b) even though the resonances only exhibit moderate Q factors (5.87 and 8.50). The inherent reason is that these plasmonic resonators are all deep-subwavelength in sizes, so that $\psi_{m}^{\text{FF}}$ and $\psi_{m}^{\text{NF}}$exhibit $\pi/2$ phase difference inside the resonator, leading to small values of $\mathrm{Im}(\Gamma_{m})$even though the strength of $\psi_{m}^{\text{FF}}$is not significantly weaker than $\psi_{m}^{\text{FF}}$(Fig. S7c).

For dielectric resonators, however, we find that the *Q*-factor required to make our theory applicable is much higher. As shown in Fig. S7e, the deviations between our theory and the spectra calculated by TMM for dielectric resonators with Q=5.88 and Q=8.56 (upper two panels in Fig. S7e) are quite obvious, manifested by non-negligible shifts in resonance (peak) frequencies. The inherent reasons are that, different from plasmonic resonators, these dielectric resonators are not naturally deep-subwavelength in sizes, so that $\psi_{m}^{\text{FF}}$ and $\psi_{m}^{\text{NF}}$do not exhibit strict $\pi/2$ phase difference inside the whole regions of resonators (see Fig. S7f). Such dielectric resonator can only exhibit a deep-subwavelength size when *n* is further enlarged (n>25), which pushes the required Q factor to an even high value (Q>20) to ensure our theory accurate enough (the lowest panel in Fig. S7e).

**References**

1. Chang, R.K. & Campillo, A.J. Optical processes in microcavities, Vol. 3. (World scientific, 1996).

2. Gerard, J.-M. in Single Quantum Dots 269-314 (Springer, 2003).

3. Novotny, L. & Hecht, B. Principles of nano-optics. (Cambridge university press, 2012).

4. Raman, A. & Fan, S. Photonic band structure of dispersive metamaterials formulated as a Hermitian eigenvalue problem. *Phys. Rev. Lett.* **104**, 087401 (2010).

5. Xi, B., Xu, H., Xiao, S. & Zhou, L. Theory of coupling in dispersive photonic systems. *Phys. Rev. B* **83**, 165115 (2011).

6. Hulst, H.C. & van de Hulst, H.C. Light scattering by small particles. (Courier Corporation, 1981).

7. Hsu, C.W., DeLacy, B.G., Johnson, S.G., Joannopoulos, J.D. & Soljacic, M. Theoretical criteria for scattering dark states in nanostructured particles. *Nano Lett.* **14**, 2783-2788 (2014).
